# The effect of proatherogenic pathogens on adipose tissue transcriptome and fatty acid distribution in apolipoprotein E-deficient mice

**DOI:** 10.1186/1471-2164-14-709

**Published:** 2013-10-17

**Authors:** Kati Hyvärinen, Anita M Tuomainen, Saara Laitinen, Georg Alfthan, Irma Salminen, Maija Leinonen, Pekka Saikku, Petri T Kovanen, Matti Jauhiainen, Pirkko J Pussinen

**Affiliations:** 1 Institute of Dentistry, University of Helsinki, P.O. Box 63, 00014 Helsinki, Finland; 2 Finnish Red Cross Blood Service, Helsinki, Finland; 3 National Institute for Health and Welfare, Helsinki, Finland; 4 National Institute for Health and Welfare, Oulu, Finland; 5 Department of Medical Microbiology, University of Oulu, Oulu, Finland; 6 Wihuri Research Institute, Helsinki, Finland; 7 FIMM, Institute for Molecular Medicine, Helsinki, Finland

**Keywords:** *A. actinomycetemcomitans*, *C. pneumoniae*, Adipose tissue, apoE-deficient mice, Transcriptome, Fatty acid distribution

## Abstract

**Background:**

Chronic infections have been demonstrated to maintain low-grade systemic inflammation and associate with atherosclerosis. We studied the inflammation- and lipid homeostasis-related effects of *Aggregatibacter actinomycetemcomitans* (Aa) and *Chlamydia pneumoniae* (Cpn) infections on the epididymal and inguinal adipose tissue (AT) transcriptomes and fatty acid distribution in apolipoprotein (apo) E-deficient mice. Chow-fed apoE-deficient mice were exposed to 1) chronic intranasal infection with *C. pneumoniae* (Cpn group), 2) recurrent intravenous infection with *A. actinomycetemcomitans* (Aa group), 3) a combination of both types of infection (Cpn + Aa group), or 4) infection with the vehicle (control group). Epididymal and inguinal AT gene expression was analyzed using an Illumina Mouse WG-6 v2.0 platform and quantitative PCR (QPCR). Microarray data were analyzed using Gene Ontology enrichment analysis. AT fatty acid analysis was performed using gas–liquid chromatography.

**Results:**

The transcriptomics data revealed significant enrichment in inflammation-associated biological pathways in both AT depots derived from the Aa and Cpn + Aa treated mice compared with the control group. The proportion of saturated fatty acids was higher in the inguinal AT in Aa (p = 0.027) and Cpn + Aa (p = 0.009) groups and in the epididymal AT in Aa group (p = 0.003). The proportion of polyunsaturated fatty acids was significantly lower among all Aa-infected groups in both depots. Chronic Cpn infection displayed only minor effects on transcriptomics and fatty acids of the AT depots.

**Conclusions:**

Systemic infection with *A. actinomycetemcomitans* activates inflammation-related biological pathways and modulates cellular lipid homeostasis. The adverse changes in adipose tissues during chronic infection may promote atherosclerosis.

## Background

Atherosclerosis is an inflammatory disease with complex multifactorial pathophysiology. In addition to the chronic inflammation in the vessel wall causing vascular endothelial dysfunction, the progression of the disease may be enhanced by persistent systemic infection, low-grade inflammation, and metabolic disturbances of major organs such as liver and adipose tissue (AT).

AT is a multi-functional metabolic organ composed of a heterogeneous cell population. By storing and releasing fatty acids and secreting several important adipokines, AT plays a significant role in both energy metabolism and homeostatic regulation of the endocrine system. The AT dysfunction, followed by the spill-over of free fatty acids (FFAs) into the circulation via enhanced lipolysis and dysregulated expression of adipokines, modifies immune responses and contributes to metabolic disorders such as metabolic syndrome and type 2 diabetes [1,2].

AT depots are dispersed throughout the body and are closely associated with several organs [3]. Recent studies have reported many site-specific physiological characteristics in AT depots, *i.e.* differences in cell populations, microvasculature, innervation, and regional variation in metabolic functions including adipokine profiles, lipase activities, uptake of fatty acids, and the insulin response [4]. These differences have a major impact on the proinflammatory potential of the various AT depots.

In humans, visceral AT has been shown to generate angiotensin, interleukin (IL) -6, and plasminogen activator inhibitor (PAI)-1 at higher levels and leptin and adiponectin at lower levels than subcutaneous AT [5]. Differences in hormone-sensitive lipase activity and maximum lipolytic capacity between omental and subcutaneous AT depots have been reported; however, these phenomena seem to be strongly correlated to the adipocyte size and their capacity to expand [6]. In obese humans, perivisceral AT has been reported to contain more saturated fatty acids (SFAs) and monounsaturated fatty acids (MUFAs) than subcutaneous AT [7]. In mice, high-fat feeding results in a continuous increase in adipocyte size both in subcutaneous and visceral fat. Interestingly, large adipocytes contain relatively more stearic acid and oleic acid, and these cells produce more tumour necrosis factor (TNF)-α, leptin, angiotensinogen, adipsin, and PAI-1 compared with small adipocytes [8].

Although AT has considerable pro-inflammatory and pro-atherogenic potential, and contributes to systemic innate immune responses, the impact of chronic systemic infections on AT is rarely investigated. Many pathogens related to common chronic infections, such as gastritis, periodontitis, and pulmonary infections, are lipopolysaccharide (LPS) containing Gram-negative species. Although LPS promotes inflammation mainly through its effects on the monocyte/macrophage function, an *in vitro* study has demonstrated that subcutaneous human adipocytes secrete large quantities of TNF-α when activated by LPS [9]. Szalowska et al. 2011 have shown that LPS activation of human omental fat results in up-regulated expression of inflammation- and angiogenesis-related genes [10].

The periodontal pathogen *Aggregatibacter actinomycetemcomitans* and the persistent pulmonary infection agent *Chlamydia pneumoniae* are Gram-negative pathogens and associated with an increased risk for cardiovascular diseases [11-13]. Both pathogens have been found in human atherosclerotic lesions [11,14], accelerate atherosclerosis in mouse model [15,16], and are highly common among general population [17,18]. We have recently shown in apolipoprotein E (apoE)-deficient mouse model that systemic *A. actinomycetemcomitans* and *C. pneumoniae* infections may induce broad range pro-atherogenic effects including alterations in macrophage cholesterol homeostasis, endothelial function [19], and, moreover, in the liver morphology, fatty acid composition, and inflammation marker profile [20].

In the present study, we investigated the effects of systemic recurrent *A. actinomycetemcomitans* and chronic *C. pneumoniae* infections on epididymal and inguinal AT depots in apoE-deficient mice. In addition, combined infection model was used to study the impact of pathogen burden on these depots. ApoE-deficient mice display a proatherogenic plasma lipoprotein profile and they develop atherosclerotic lesions on a chow diet. In addition, due to decreased levels of LPS-neutralizing high-density lipoproteins, apoE-deficient mice show increased susceptibility to LPS-mediated inflammation compared to wild-type mice [21]. In order to include both visceral and subcutaneous AT and to identify their putative depot-specific pro-inflammatory and pro-atherogenic potential, we utilized both epididymal and inguinal AT depots. We focused on molecules associated with inflammation and lipid homeostasis, which were investigated on the level of gene expression and cellular fat composition.

## Methods

### Mice

Male apolipoprotein (apo) E-deficient mice (B6.129P2- Apoe^tm1Unc^/Crl) were purchased from Charles River Laboratories. The mice were fed regular mouse chow *ab libitum* and maintained in a germ-free environment at the National Public Health Institute Animal Facilities (Helsinki, Finland). Animal care and experimentation were conducted under Animal Care and Use Committee of National Public Health Institute ethical authorization and were in accordance with the guidelines of the Council of Europe.

### Bacterial cultures, infection procedure, and serum analyses

The bacteria cultures and experimental design have been previously reported [20]. Briefly, the clinical *A. actinomycetemcomitans* strain AT445b (serotype b) was cultivated in a tryptic soy-serum-bacitracin-vancomycin medium at 37°C with 5% CO_2_ for 48 h. The *C. pneumoniae* isolate, Kajaani 7, a Finnish epidemic strain, was isolated and cultured as previously described [22].

The infection procedure is presented in Table 1. The experimental design included chronic *C. pneumoniae* infection model, which was achieved by three consecutive inoculations, recurrent *A. actinomycetemcomitans* infection model mimicking repeated systemic exposure to the pathogen, and a combination of these two infection models to imitate the effects of infection burden. Chronic *C. pneumoniae* infection was induced in younger mice e in order to see the potential effect of persistence. Viable *C. pneumoniae* (Cpn) was administered intranasally as 2 × 10^6^ inclusion forming units in 40 μl of sucrose-phosphate-glutamic acid buffer (SPG), and viable *A. actinomycetemcomitans* (Aa) was administered intravenously (via the tail vein) as 1 × 10^7^ colony forming units in 50 μl of 0.9% NaCl. The 10-week experiment included four groups: 1) recurrent Aa infection group (Aa group, n = 10) with ten Aa inoculations; 2) the chronic Cpn infection group (Cpn group, n = 10) with three Cpn inoculations; 3) the combined chronic Cpn and recurrent Aa infection group (Cpn + Aa group, n = 10) with three Cpn inoculations and ten Aa inoculations; and 4) the control group (n = 9) with three SPG inoculations and ten 0.9% NaCl inoculations.

**Table 1:** Experimental design of the study

**Study group**	**Age (weeks)**															
	**9**	**10**	**11**	**12**	**13**	**14**	**15**	**16**	**17**	**18**	**19**	**20**	**21**	**22**	**23**	**24**
**Cpn group**	▪		▪		▪											†
**Aa group**						●	●	●	●	●	●	●	●	●	●	†
**Cpn + Aa group**	▪		▪		▪	●	●	●	●	●	●	●	●	●	●	†
**Control**	□		□		□	○	○	○	○	○	○	○	○	○	○	†

The black squares represent Cpn inoculations and the black circles Aa inoculations. The controls received the respective vehicle (white squares and circles). †, sacrifice; Aa, *A. actinomycetemcomitans*; Cpn, *C. pneumoniae.*

The serum levels of nonesterified fatty acids (Waco Chemicals GmbH), the LPS activity (HyCult biotechnology b.v.), and the serum levels of TNFα (Pierce Biotechnology, Inc.) were measured as described in our previous publications [19,20].

### Preparation of adipose tissues

Inguinal and epididymal AT depots were carefully dissected from sacrificed animals. The tissue specimens were stabilized with RNA*later*™ (Sigma) or snap-frozen in liquid nitrogen. All the samples were stored at -70°C.

### Isolation and quality evaluation of RNA

Epididymal and inguinal AT homogenization was performed with a Mixer Mill MM 301 (Retsch GmbH) and total RNA was extracted using the RNeasy Lipid Tissue Mini Kit (Qiagen). DNase treatment was performed on a column using the RNase-Free DNase Set (Qiagen) and again after RNA extraction using the DNA-free™ Kit (Ambion). The RNA concentration was determined using a NanoDrop 1000 spectrophotometer (Thermo Scientific). The quality of total RNA was assessed by an Agilent 2100 Bioanalyzer using the Eukaryote Total RNA Nano Kit (Agilent Technologies).

### Adipose tissue RNA microarray processing

Inguinal and epididymal AT gene expression was analyzed using the Illumina Mouse WG-6 v2.0 platform. Three biological replicates were used and the high quality total RNA (RNA integrity number ≥ 8.5) was amplified using the Illumina TotalPrep RNA Amplification Kit according to the manufacturer’s instructions. Briefly, the total RNA was reverse-transcribed using T7 Oligo(dT) primers following the second strand cDNA synthesis. After purification, the cDNA was *in vitro* transcripted into cRNA using biotin-labeled nucleoside triphosphates. The quality of biotinylated cRNA was assessed by an Agilent 2100 Bioanalyzer and 1.5 μg of cRNA from each sample was hybridized at 58°C overnight using the Illumina Whole-Genome Gene Expression Direct Hybridization Assay system with the six-sample BeadChip platform. The signal was developed using streptavidin-Cy3 and the BeadChips were scanned with an Illumina BeadArray Reader.

### Microarray data analyses

The microarray data has been deposited in MIAME-compliant format in Gene Expression Omnibus (http://www.ncbi.nlm.nih.gov/geo/), accession number GSE50647. Gene ontology (GO) enrichment analysis for microarray data was performed using the Fisher's exact test, where the observed frequency of each present GO term was compared with the frequency in a reference gene set (control mice). P-values were corrected for multiple hypotheses using the Benjamini-Hochberg false discovery rate [23]. The analyses were performed using the R Base Package version 2.15.0. The maximum of 20 most enriched GO terms were reported for both epididymal and inguinal AT and ranked according to the p-value. To limit the data and focus on the biologically meaningful pathways and molecular functions, GO terms referring to cellular components were excluded. The fold-change (FC) limitation for the presented up- and down-regulated genes was 2.0 except when observing *de novo* lipogenesis-associated genes where all results are present.

### Quantitative real-time PCR

Epididymal and inguinal AT cDNA was synthesized from total RNA using the ImProm-II™ Reverse Transcription system (Promega). The amount of total RNA in each reverse transcription (RT) reaction was 240 ng for the inguinal AT and 500 ng for the epididymal AT. The number of mice in each group was 10, except for the control group where 9 mice were used. Primers (Thermo Scientific) were designed using Beacon Designer (Premier Biosoft International) and National Center for Biotechnology Information Primer-BLAST (http://www.ncbi.nlm.nih.gov/tools/primer-blast/) for CD68, glyceraldehyde-3-phosphate dehydrogenase (Gapdh), monocyte chemoattractant protein 1 (Mcp-1), macrophage migration inhibitory factor (Mif), mannose receptor C type 1 (Mrc-1), myeloperoxidase (Mpo), and secretory leukocyte peptidase inhibitor (Slpi). Primer sequences are presented in Additional file 1: Table S1. Quantitative real-time PCR (QPCR) was performed using the Mx3005 Real-Time QPCR System (Stratagene). The analyses were conducted in 12.5 μl reaction mixtures, each containing 2 μl of 1:50 dilution of cDNA synthesized in RT reaction, 6.25 μl of Brilliant SYBR® Green QPCR Master Mix (Stratagene), 30 nM of ROX reference dye (Stratagene), and optimized concentrations of primers (100 nM, except 200 nM for Gapdh). The amplifications were performed using the following thermocycling program: initial denaturation at 95°C for 15 min, 40 cycles of 15 s at 95°C and 1 min at 60°C followed by melting curve analysis for 1 min at 95°C, gradual decrease to 55°C, 30 s at 55°C, gradual increase to 95°C, and 30 s at 95°C. All samples were analyzed as duplicates and the accepted intra-assay coefficient of variation for threshold cycle was ≤ 2.1%. The results were analyzed using Mx3005 Real-Time QPCR System (Stratagene) software and expressed as a log_2_ fold change (log_2_FC) to the respective control mice (level set to zero in the analysis). Gapdh was used as a normalization gene and the software utilizes modified comparative quantitation method introduced by Pfaffl (2001) [24].

### Adipose tissue fatty acid analysis

Total lipids were extracted from 30–80 mg of epididymal and inguinal adipose tissue with hexane-isopropanol (3:2) [25] and trans-methylated by heating with acidic methanol (5% H_2_SO_4_) [26]. The resulting fatty acid methyl ester compositions were analyzed by gas–liquid partition chromatography using a Hewlett Packard 6890 Gas Chromatograph (ChemStation B.01.03 software) with a DB-225 GC column (30 m, diameter 0.32 mm, phase layer 0.25 μm; Agilent Technologies). The samples were split injected with hydrogen as a carrier gas and the temperature program was from 160°C to 230°C. The relative amounts of the methylated fatty acids were expressed as percentages of weight normalized to 100%.

### Statistical analyses

The AT fatty acid data were expressed as mean percentages of weight with standard deviations (SDs). The gene expression data were expressed as log_2_ fold change (log_2_FC) medians with interquartile ranges (IQRs). The significance of the differences between the experimental and control mouse groups was assessed using the non-parametric Mann–Whitney *U* test. Bivariate correlations were determined using the Spearman test. Analyses were performed using PASW Statistics 18 (Statistical Package for the Social Sciences).

## Results

### Infection-enriched biological pathways in the inguinal AT

Infection-enriched biological pathways (*i.e*., GO terms) and the associated up- and down-regulated genes in the inguinal AT transcriptome are presented in Tables 2, 3 and 4. Compared with the control mice, the expression of “Protein binding” pathway (GO:0005515) was increased (p <0.001) most significantly in Aa group (Table 2). In addition, the inflammation-related “Antigen binding” (GO:0003823, p = 0.009), “Antibacterial humoral response” (GO:0019731, p = 0.009), and “Antimicrobial humoral response” (GO:0019730, p = 0.009) pathways were significantly enriched (Table 2).

**Table 2:** ***A. actinomycetemcomitans *** **infection-enriched GO terms in the inguinal AT transcriptome**

**GO category**	**Enriched GO term**	**GO term p-value**^**a**^	**GO term-associated gene products**	
			**Up-regulated**^**b**^	**Down-regulated**^**b**^
GO:0005515	Protein binding	<0.001	Spon2, S100a8, Ltf, Des, Retnlg, D6Mit97, LOC100047788, Igl, Igh-V11, Ighg3, Igkv12-46, Igkv5-48, Igkv13-84, Igkv19-93, Igkv15-103	Zbtb7a, Rhob, Aldh1a1, Ywhag, Ccdc6, Ptplb, Antxr1, Timp4, Rps3a, Ddr2, Cidea, Nr1d2, G0s2, Lep, Acta1
GO:0019731	Antibacterial humoral response	0.009	LOC100047788	-
GO:0019730	Antimicrobial humoral response	0.009	LOC100047788	-
GO:0003823	Antigen binding	0.009	Igkv5-48, LOC100047788	-

^a^P-values are corrected for multiple hypotheses using Benjamini-Hochberg false discovery rate.

^b^Fold change limit 2.0.

Number of biological replicates is 3.

GO, gene ontology. The maximum of 20 GO categories is shown.

**Table 3:** ***C. pneumoniae *** **infection-enriched GO terms in the inguinal AT transcriptome**

**GO category**	**Enriched GO term**	**GO term p-value**^**a**^	**GO term-associated gene products**	
			**Up-regulated**^**b**^	**Down-regulated**^**b**^
GO:0035634	Response to stilbenoid	<0.001	Ifit3, Usp18	-
GO:0019785	ISG15-specific protease activity	<0.001	Usp18	-
GO:0032020	ISG15-protein conjugation	<0.001	Usp18	-
GO:0001823	Mesonephros development	0.001	Osr2, Gpc3	-
GO:0033687	Osteoblast proliferation	0.002	Osr2, Eif2ak2	-
GO:0035116	Embryonic hindlimb morphogenesis	0.003	Osr2, Gpc3	-
GO:2000543	Positive regulation of gastrulation	0.008	Osr2	-
GO:0061029	Eyelid development in camera-type eye	0.008	Osr2	-
GO:0060272	Embryonic skeletal joint morphogenesis	0.008	Osr2	-
GO:0048793	Pronephros development	0.008	Osr2	-
GO:0030282	Bone mineralization	0.013	Osr2, Gpc3	-
GO:0001656	Metanephros development	0.013	Osr2, Gpc3	-
GO:0042474	Middle ear morphogenesis	0.020	Osr2	-
GO:0004221	Ubiquitin thiolesterase activity	0.020	Usp18	-
GO:0035115	Embryonic forelimb morphogenesis	0.030	Osr2	-
GO:0030501	Positive regulation of bone mineralization	0.030	Osr2	-
GO:0048523	Negative regulation of cellular process	0.036	Slfn1, Gpc3, Ppap2b, Eif2ak2, Osr2	Acaa2, Ddit4
GO:0030163	Protein catabolic process	0.042	Usp18, Gpc3	-
GO:0050678	Regulation of epithelial cell proliferation	0.042	Osr2, Gpc3	-
GO:0043401	Steroid hormone mediated signaling pathway	0.042	-	Nr1d2

^a^P-values are corrected for multiple hypotheses using Benjamini-Hochberg false discovery rate.

^b^Fold change limit 2.0.

Number of biological replicates is 3.

GO, gene ontology. The maximum of 20 GO categories is shown.

**Table 4:** **Combined chronic *C. pneumoniae *** **and recurrent *A. actinomycetemcomitans *** **infections-enriched GO terms in the inguinal AT transcriptome**

**GO category**	**Enriched GO term**	**GO term p-value**^**a**^	**GO term-associated gene products**	
			**Up-regulated**^**b**^	**Down-regulated**^**b**^
GO:0005515	Protein binding	<0.001	Igk-V5, Igkv5-48, Igkv4-80, Igkv8-30, IgkV33, Igkv12-89, Igkv15-103, Igkv2-112, D6Mit97, S100a8, Ltf, LOC637260, LOC626347, Igl, Ighv1-62, Ighv6-3, LOC630305, Ighg3, Igh-4,Ighg, LOC100047788, Gm16971, Ngp, Chi3l3	Aldh1a1, Adh7, Ddx17, Acta1, Bmp3, Igfbp6, Tnnc2, Lep, Dpt
GO:0003823	Antigen binding	<0.001	LOC100047788, Igkv5-48, Igkv2-112	-
GO:0019731	Antibacterial humoral response	0.002	LOC100047788	-
GO:0019730	Antimicrobial humoral response	0.002	LOC100047788	-
GO:0051952	Regulation of amine transport	0.002	-	Lep, Sncg
GO:0042572	Retinol metabolic process	0.011	-	Aldh1a1, Adh7
GO:0030534	Adult behavior	0.011	-	Lep, Sncg
GO:0014059	Regulation of dopamine secretion	0.011	-	Sncg
GO:0014046	Dopamine secretion	0.011	-	Sncg
GO:0006836	Neurotransmitter transport	0.011	-	Slc6a13, Sncg
GO:0042573	Retinoic acid metabolic process	0.013	-	Aldh1a1, Adh7
GO:0002455	Humoral immune response mediated by circulating immunoglobulin	0.016	LOC100047788	-
GO:0046928	Regulation of neurotransmitter secretion	0.020	-	Sncg
GO:0008344	Adult locomotory behavior	0.042	-	Sncg
GO:0032526	Response to retinoic acid	0.044	-	Aldh1a1, Lep
GO:2000366	Positive regulation of STAT protein import into nucleus	0.046	-	Lep
GO:0071298	Cellular response to L-ascorbic acid	0.046	-	Lep
GO:0060587	Regulation of lipoprotein lipid oxidation	0.046	-	Lep
GO:0051956	Negative regulation of amino acid transport	0.046	-	Lep
GO:0034439	Lipoprotein lipid oxidation	0.046	-	Lep

^a^P-values are corrected for multiple hypotheses using Benjamini-Hochberg false discovery rate.

^b^Fold change limit 2.0.

Number of biological replicates is 3.

GO, gene ontology. The maximum of 20 GO categories is shown.

Compared with the control mice, most of the enriched GO terms in the inguinal AT transcriptome of Cpn group were pathways associated with development- and morphogenesis (Table 3). The most affected pathway was “Negative regulation of cellular process” pathway (GO:0048523, p = 0.036).

The most significantly enriched pathway in the inguinal AT transcriptome of Cpn + Aa group was “Protein binding” (GO:0005515, p <0.001) followed by inflammation-related “Antigen binding” (GO:0003823, p <0.001), “Antibacterial humoral response” (GO:0019731, p = 0.002), and “Antimicrobial humoral response” (GO:0019730, p = 0.002) pathways (Table 4). Lipid homeostasis-related “Regulation of lipoprotein lipid oxidation” (GO:0060587, p = 0.046) and “Lipoprotein lipid oxidation” (GO:0034439, p = 0.046) pathways were also enriched.

### Infection-enriched biological pathways in the epididymal AT

Table 5 presents the Aa infection-enriched biological pathways in the epididymal AT transcriptome. Compared with the control mice, in the epididymal AT, infection enriched the following humoral antibody response and inflammation-associated pathways: “Antigen binding” (GO:0003823, p < 0.001), “Antibacterial humoral response” (GO:0019731, p = 0.002), “Antimicrobial humo-ral response” (GO:0019730, p = 0.002), “Immunoglobulin mediated immune response” (GO:0016064, p = 0.004), “Humoral immune response mediated by circulating immunoglobulin” (GO:0002455, p = 0.013), and “Inflammatory response” (GO:0006954, p = 0.049). The lipid homeostasis-related “Monocarboxylic acid metabolic process” (GO:0032787, p = 0.006), “Fatty acid biosynthetic process” (GO:0006633, p = 0.006), “Regulation of lipid metabolic process” (GO:0019216, p = 0.012), “Negative regulation of lipid metabolic process” (GO:0045833, p = 0.015), “Triglyceride metabolic process” (GO:0006641, p = 0.027), and “Regulation of lipid biosynthetic process” (GO:0046890, p = 0.032) were also enriched.

**Table 5:** ***A. actinomycetemcomitans *** **infection-enriched GO terms in the epididymal AT transcriptome**

**GO category**	**Enriched GO term**	**Go term p-value**^**a**^	**GO term-associated gene products**	
			**Up-regulated**^**b**^	**Down-regulated**^**b**^
GO:0003823	Antigen binding	<0.001	Igk-C, LOC100047788	-
GO:0019731	Antibacterial humoral response	0.002	LOC100047788	-
GO:0019730	Antimicrobial humoral response	0.002	LOC100047788	-
GO:0016064	Immunoglobulin mediated immune response	0.004	LOC100047788	Bcl6
GO:0032787	Monocarboxylic acid metabolic process	0.006	Pcx, Insig1, Elovl6, Scd2	-
GO:0006633	Fatty acid biosynthetic process	0.006	Insig1, Elovl6, Scd2	-
GO:0019216	Regulation of lipid metabolic process	0.012	Cidea, Insig1, Thrsp	-
GO:0002455	Humoral immune response mediated by circulating immunoglobulin	0.013	LOC100047788	-
GO:0045833	Negative regulation of lipid metabolic process	0.015	Cidea, Insig1	-
GO:0005515	Protein binding	0.022	Slc15a2, Cidea, Thrsp, Elovl6, Lrtm1, Igk-V5, LOC384413, LOC637227, Insig1, LOC100047162, LOC669053, Igk-C, LOC100047628, LOC100047788, Ighg, Ighg3, LOC676136	Bcl6, Itga11
GO:0006641	Triglyceride metabolic process	0.027	Thrsp, Insig1	-
GO:0046890	Regulation of lipid biosynthetic process	0.032	Thrsp, Insig1	-
GO:0009611	Response to wounding	0.034	Fabp4, Klf6	Chst4, Bcl6
GO:0001816	Cytokine production	0.045	Fabp4, Cidea	Bcl6
GO:0001818	Negative regulation of cytokine production	0.046	Cidea	Bcl6
GO:0051973	Positive regulation of telomerase activity	0.048	9130213B05Rik	-
GO:0042938	Dipeptide transport	0.048	Slc15a2	-
GO:0006954	Inflammatory response	0.049	Fabp4	Chst4, Bcl6

^a^P-values are corrected for multiple hypotheses using Benjamini-Hochberg false discovery rate.

^b^Fold change limit 2.0.

Number of biological replicates is 3.

GO, gene ontology. The maximum of 20 GO categories is shown.

Table 6 presents the Cpn infection-enriched pathways in the epididymal AT transcriptome. The enriched pathways included “Very long-chain fatty acid metabolic process” (GO:0000038, p = 0.003), “Long-chain fatty acid metabolic process” (GO:0001676, p = 0.003), “Long-chain fatty acid-CoA ligase activity” (GO:0004467, p <0.001), and “Very long-chain fatty acid-CoA ligase activity” (GO:0031957, p <0.001) pathways. The most affected pathway was “Defense response” (GO:0006952, p = 0.003).

**Table 6:** ***C. pneumoniae *** **infection-enriched GO terms in the epididymal AT transcriptome**

**GO category**	**Enriched GO term**	**Go term p-value**^**a**^	**GO term-associated gene products**	
			**Up-regulated**^**b**^	**Down-regulated**^**b**^
GO:0009206	Purine ribonucleoside triphosphate biosynthetic process	<0.001	Nme7, Atp1b1, Ldhc,1600029I14Rik	-
GO:0006754	ATP biosynthetic process	<0.001	Nme7, Atp1b1, Ldhc	-
GO:0005391	Sodium:potassium-exchanging ATPase activity	<0.001	Nme7, Atp1b1	-
GO:0031957	Very long-chain fatty acid-CoA ligase activity	<0.001	Acsbg1, Slc27a2	-
GO:0030317	Sperm motility	<0.001	Spag6, Tnp1, Ldhc, Smcp	-
GO:0004467	Long-chain fatty acid-CoA ligase activity	<0.001	Acsbg1, Slc27a2	-
GO:0006952	Defense response	0.003	Ccl8, Arg2, Defb22, Cx3cl1, Nt5e, Defb29, Defb36, Defb38, Cfi, Elf3	Ccl5, Orm1, Orm2, S100a8, Ccl11
GO:0001676	Long-chain fatty acid metabolic process	0.003	Acsbg1, Slc27a2	-
GO:0000038	Very long-chain fatty acid metabolic process	0.003	Acsbg1, Slc27a2	-
GO:0006323	DNA packaging	0.004	Sox9, Prm1	Hist1h4i, Hist2h2ac, Hist1h4k, Hist1h4j, Hist1h3d
GO:0016338	Calcium-independent cell-cell adhesion	0.005	Cldn2, Cldn11, Cx3cl1	
GO:0009991	Response to extracellular stimulus	0.007	Slc39a4, Mt3, Srd5a2, Sox9, Lrp2, Sfrp1, Arg2, Avpr1a	Tnfrsf11b, Ccl5, Acta1
GO:0034728	Nucleosome organization	0.008	Sox9, Tnp1	Hist1h3d, Hist2h2ac, Hist1h4i, Hist1h4k, Hist1h4j
GO:0071345	Cellular response to cytokine stimulus	0.008	Mt3, Cx3cl1, Krt18, Sox9, Krt8, Sfrp1, Arg2	Ccl5
GO:0042493	Response to drug	0.008	Slc15a2, Mt3, Srd5a2, Lrp2, Sfrp1, Arg2, Slc22a1	Tnfrsf11b, Ccl5, Cox8b
GO:0048245	Eosinophil chemotaxis	0.009	LOC100048554	Ccl5, Ccl11
GO:0022414	Reproductive process	0.009	Spag6, Sox9, 4931407G18Rik, Avpr1a, Arg2, Prm1, Tnp1, Odf2, Smcp, Acsbg1, Cldn11, Srd5a2, Mycbpap, Sfrp1	Ccl5
GO:0006333	Chromatin assembly or disassembly	0.009	Sox9, Tnp1	Hist1h3d, Hist2h2ac, Hist1h4i, Hist1h4k, Hist1h4j,
GO:0048609	Multicellular organismal reproductive process	0.010	4931407G18Rik, Avpr1a, Arg2, Prm1, Spag6, Smcp, Odf2, Sfrp1, Cldn11, Mycbpap, Sox9, Tnp1	-
GO:0032504	Multicellular organism reproduction	0.010	Sox9, 4931407G18Rik, Arg2, Prm1, Spag6, Avpr1a, Odf2, Sfrp1, Cldn11, Tnp1, Mycbpap, Smcp	-

^a^P-values are corrected for multiple hypotheses using Benjamini-Hochberg false discovery rate.

^b^Fold change limit 2.0.

Number of biological replicates is 3.

GO, gene ontology. The maximum of 20 GO categories is shown.

Table 7 presents the Cpn + Aa infections-enriched pathways in the epididymal AT transcriptome. The “Protein binding” pathway (GO:0005515, p <0.001) included the highest number of affected genes. Inflammation-related “Antigen binding” (GO:0003823, p < 0.001), “Antibacterial humoral response” (GO:0019731, p = 0.016), and “Antimicrobial humoral response” (GO:0019730, p = 0.016) pathways were enriched. There were no enrichments in lipid metabolism-related pathways.

**Table 7:** **Combined chronic *C. pneumoniae *** **and recurrent *A. actinomycetemcomitans *** **infections-enriched GO terms in the epididymal AT transcriptome**

**GO category**	**Enriched GO term**	**Go term p-value**^**a**^	**GO term-associated gene products**	
			**Up-regulated**^**b**^	**Down-regulated**^**b**^
GO:0045653	Negative regulation of megakaryocyte differentiation	<0.001	-	Hist1h4h, Hist1h4i, Hist1h4k, Hist1h4j
GO:0006334	Nucleosome assembly	<0.001	Sox9	Hist1h4j, Hist1h4i, Hist1h4k, Hist1h4h, Hist1h3d, Hist2h2ac
GO:0005515	Protein binding	<0.001	BC021891, Lrrc48, Gja1, Cldn2, Lrtm1, Pcp4l1, Cldn11, Ubd, Dynlrb2, Mt3, Clic6, Sh3gl2, Lrp2, Nme7, Slc40a1, Cxcl13, Fcgr4, Spag6, Ccl8, Sox9, Alox12, Sfrp1, Ighv1-62, Col6a5, C7, Ighg3, Ighg, LOC100047788, Igk-C, D6Mit97, Igk-V5, Igkv12-46, LOC384419, LOC637227, Igkv4-68	Itga11, Acta1, Bmp3, Sfrp5, Hist1h4i, Hist1h4h, Hist1h4k, Wdr92, Hist1h3d, Hist1h4j
GO:0003823	Antigen binding	<0.001	LOC100047788, Igk-C	-
GO:0016337	Cell-cell adhesion	0.004	Sox9, Cxcl13, Cldn11, 1110049B09Rik, Cldn2, Dsg2	Chst4
GO:0016338	Calcium-independent cell-cell adhesion	0.011	Cldn2, Cldn11	-
GO:2000117	Negative regulation of cysteine-type endopeptidase activity	0.016	Sfrp1, Mt3	-
GO:0019731	Antibacterial humoral response	0.016	LOC100047788	-
GO:0019730	Antimicrobial humoral response	0.016	LOC100047788	-
GO:0008219	Cell death	0.016	Mt3, Alox12, Sox9, Slc40a1, Sfrp1, Acsbg1, Mmd2, Ubd, Bex2, Dsg2, Gja1	Lyz1, Wdr92
GO:0071504	Cellular response to heparin	0.021	Sfrp1, Sox9	-
GO:0006352	Transcription initiation, DNA-dependent	0.021	-	Hist1h4h, Hist1h4i, Hist1h4k, Hist1h4j
GO:0019835	Cytolysis	0.024	Mmd2	Lyz1
GO:0008253	5′-nucleotidase activity	0.041	Nt5e, Acpp	-

^a^P-values are corrected for multiple hypotheses using Benjamini-Hochberg false discovery rate.

^b^Fold change limit 2.0.

Number of biological replicates is 3.

GO, gene ontology. The maximum of 20 GO categories is shown.

### Inflammation- and lipid homeostasis-related up- and down-regulated genes in the inguinal AT

In Aa group, most of the “Protein binding” pathway-associated up-regulated genes belonged to immunoglobulin variables (Table 2, Additional file 2: Table S2). The most up-regulated genes were S100 calcium binding protein A8 (*S100a8*, FC = 13.27), S100 calcium binding protein A9 (*S100a9*, FC = 9.45), and lactotransferrin (*Ltf*, FC = 6.62). Lipid homeostasis-associated leptin (*Lep*, FC = 0.46) was down-regulated (Additional file 2: Table S2).

In Cpn group, infection induced down-regulation of acetyl-Coenzyme A acyltransferase 2 (*Acaa2*, FC = 0.41), ELOVL family member 6, elongation of long chain fatty acids (*Elovl6*, FC = 0.44), and nuclear receptor subfamily 1, group D, member 2 (*Nr1d2*, FC = 0.44) (Additional file 3: Table S3).

In Cpn + Aa group, the majority of up-regulated genes were immunoglobulin variables (Additional file 4: Table S4). In addition, *S100a9* (FC = 11.29), *S100a8* (FC = 10.96), and *Ltf* (FC = 4.73) were up-regulated. *Lep* (FC = 0.46) and synuclein δ (Sncg, FC = 0.040) were down-regulated (Additional file 4: Table S4).

### Inflammation- and lipid homeostasis-related up- and down-regulated genes in the epididymal AT

In Aa group, the enriched “Monocarboxylic acid metabolic process” pathway (p = 0.006) included four up-regulated genes: pyruvate carboxylase (*Pcx*, FC = 2.82), *Elovl6* (FC = 3.21), stearoyl-coenzyme A desaturase 2 (*Scd2*, FC = 2.15), and insulin induced gene 1 (*Insig1*, FC = 2.01) (Additional file 5: Table S5). *Insig1*, cell death-inducing DNA fragmentation factor, alpha subunit-like effector A (*Cidea*, FC = 2.53) and thyroid hormone responsive (*Thrsp*, FC = 2.26) were associated with the “Regulation of lipid metabolic process” pathway (p = 0.012). In addition, fatty acid binding protein 4 (*Fabp4*, FC = 2.60) and immunoglobulin variables were up-regulated (Additional file 5: Table S5).

In Cpn group, the “Defense response” pathway (p = 0.003) included four defensin subtypes, 5′ nucleotidase, ecto (*Nt5e*, FC = 3.34), and chemokine (C-C motif) ligand 8 (*Ccl8*, FC = 2.81) (Additional file 6: Table S6). The up-regulated solute carrier family 27 (fatty acid transporter), member 2 (*Slc27a2*, FC = 3.09) was associated with the lipid homeostasis-related pathways.

In Cpn + Aa group, a large number of up-regulated genes were immunoglobulin variables and the most up-regulated was Ig gamma-2A chain C region secreted form-like (LOC100047788, FC = 37.73) (Additional file 7: Table S7). Lipid homeostasis -associated arachidonate 12-lipoxygenase (*Alox12*, FC = 3.91) was also up-regulated.

### The expression of *de novo* lipogenesis-associated genes

**Figure 1:**
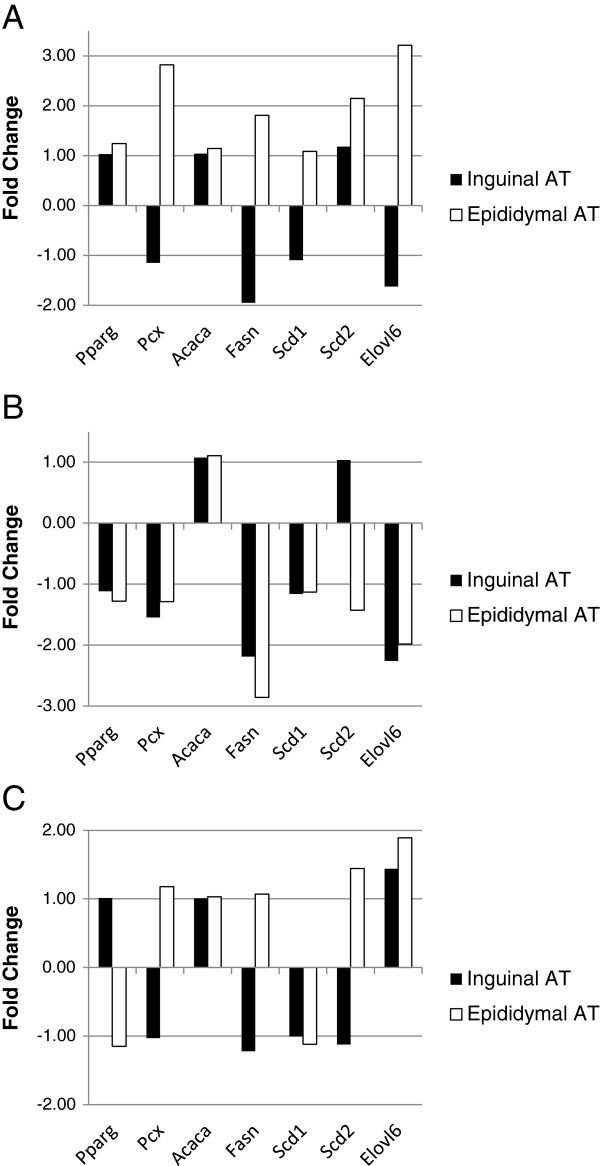
**The expression of *de novo *lipogenesis associated genes among different AT depots and infection models.** The gene expression fold changes were calculated from the microarray analysis data as described in the Methods section. Differences in gene expression in the inguinal and epididymal AT transcriptomes induced by **A)** recurrent *A. actinomycetemcomintas* infection, **B)** chronic *C. pneumoniae* infection and **C)** combined recurrent *A. actinomycetemcomintas* and *C. pneumoniae* infections. The changes were statistically non-significant after false discovery rate correction. *Acaca*, acetyl-Coenzyme A carboxylase alpha; *Elovl6*, ELOVL family member 6, elongation of long chain fatty acids (yeast); *Fasn*, fatty acid synthase; *Pcx*, pyruvate carboxylase; *Pparg*, peroxisome proliferator activated receptor γ; *Scd1*, stearoyl-Coenzyme A desaturase 1; *Scd2*, stearoyl-Coenzyme A desaturase 2.

Figure 1 presents the expression of *de novo* lipogenesis -associated genes in the AT depot transcriptomes among the different infection models (the data are derived from the microarray experiment). In the epididymal transcriptome of Aa-infected mice, the up-regulated genes were *Pcx* (FC = 2.82), fatty acid synthase (*Fasn*, FC = 1.81), *Scd2* (FC = 2.15), and *Elovl6* (FC = 3.21) (Figure 1A). *Fasn* and *Elovl6* were down-regulated in the Cpn group in both AT depots (inguinal *Fasn* FC = -2.19, epididymal *Fasn* FC = -2.86; inguinal *Elovl6* FC = -2.26, epididymal *Elovl6* FC = -1.98) (Figure 1B). The combined infection model had little impact on *de novo* lipogenesis associated gene expression (Figure 1C).

### Alterations in QPCR-analyzed mRNA expression levels

The relative mRNA expression levels of neutrophil activation- (*Slpi* and *Mpo*) and macrophage phenotype- (macrophage type 2 marker *Mrc-1*) and activation- (*Mcp-1* and *Mif*) related genes in inguinal and epididymal AT are presented in Table 8. In the inguinal AT, the relative expression levels of genes encoding *Slpi* and *Mpo* were significantly higher in the Aa group (*Slpi*: p = 0.003; *Mpo*: p = 0.009) than in control mice (level set to zero in the analysis). Cpn infection did not have a significant effect on the selected gene expression levels.

**Table 8:** Relative changes in mRNA expression levels of selected genes in inguinal and epididymal AT

		**Aa group**	**Cpn group**	**Cpn + Aa group**
		**(n = 10)**	**(n = 10)**	**(n = 10)**
**Tissue**	**Gene**	**Median Log**_**2 **_**fold change (IQRs)**^**a**^		
		**p-value**^**b**^		
**Inguinal AT**	***Mcp-1***	-0.78 (1.96)	-0.31 (1.44)	-0.53 (1.76)
		0.671	0.348	0.020
	***Mrc-1***	-0.19 (1.05)	-0.17 (1.00)	0.02 (2.08)
		0.671	1.000	1.000
	***CD68***	0.44 (1.56)	0.25 (1.23)	0.03 (1.36)
		0.203	0.061	0.437
	***Mif***	0.57 (2.15)	0.40 (1.94)	-0.11 (1.67)
		0.203	0.348	1.000
	***Slpi***	4.73 (2.99)	-1.18 (3.55)	2.79 (6.50)
		0.003	0.061	0.437
	***Mpo***	4.30 (2.64)	-0.48 (3.75)	4.04 (7.01)
		0.009	0.115	0.348
**Epididymal AT**	***Mcp-1***	-0.73 (1.97)	-0.41 (1.88)	-1.03 (1.46)
		0.020	0.203	<0.001
	***Mrc-1***	-0.16 (0.88)	0.60 (0.53)	-0.17 (0.99)
		0.437	0.003	0.599
	***CD68***	-0.39 (0.90)	0.58 (0.76)	0.55 (1.86)
		0.120	0.034	0.600
	***Mif***	-0.88 (0.70)	-0.13 (1.06)	-0.86 (0.67)
		0.020	0.203	<0.001
	***Slpi***	-0.89 (2.11)	0.20 (2.20)	-0.24 (2.07)
		0.002	0.203	0.600

^a^Median Log_2_ fold change (IQRs) to the control mice. The control mice have been set into the value of 0.

^b^The Mann–Whitney *U* Test with the control mice as the reference group.

Aa group, recurrent *A. actinomycetemcomitans* infection; Cpn group, chronic *C. pneumoniae* infection; Cpn + Aa group, combined recurrent *A. actinomycetemcomitans* and chronic *C. pneumoniae* infections; IQR, interquartile range; *Mcp-1*, monocyte chemoattractant protein 1; *Mif*, macrophage migration inhibitory factor, *Mrc-1*, mannose receptor C type 1; Mpo, myeloperoxidase; *Slpi*, secretory leukocyte peptidase inhibitor.

In the epididymal AT, the relative mRNA expression levels of *Mcp-1*, *Mif*, and *Slpi* in the Aa group, *Mrc-1* and CD68 in Cpn group, and *Mif* in Aa + Cpn group were significantly altered but between -1 to 1 log_2_FC . The Cpn + Aa combination induced a reduction in the *Mcp-1* mRNA levels (p <0.001).

### Changes in AT fatty acid composition

**Figure 2:**
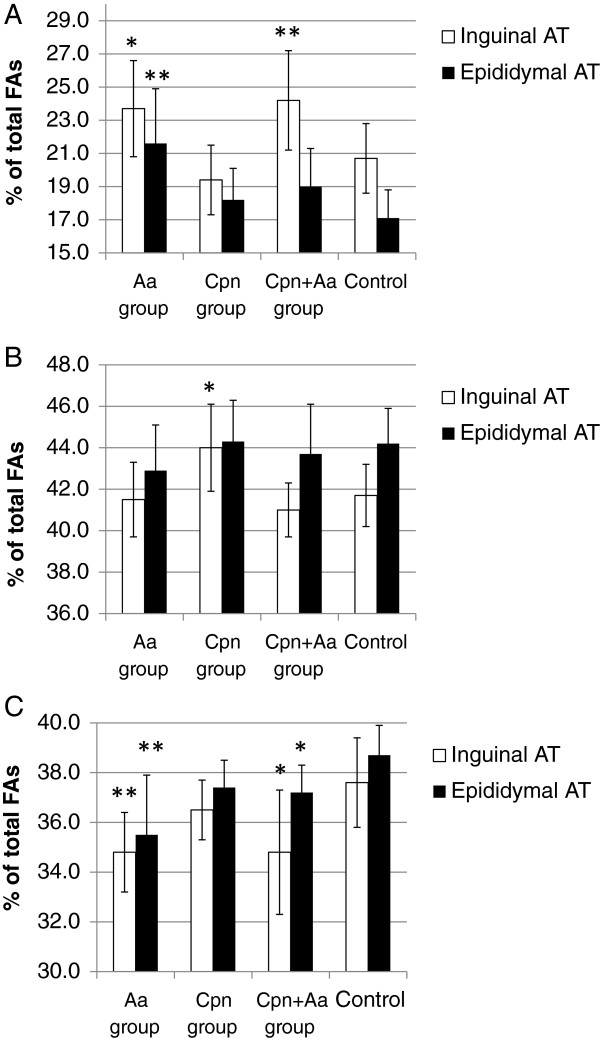
**Proportions of fatty acids in inguinal and epididymal AT.** The AT FAs were analyzed by gas liquid chromatography as described in the Methods section. The results are expressed as percentage of weight normalized to 100%. Proportions of **A)** SFAs, **B)** MUFAs, and **C)** PUFAs in inguinal and epididymal AT. The statistical significance of differences was determined with Mann–Whitney *U* Test using the respective control group as the reference group. *, p <0.05; ** p <0.01; AT, adipose tissue; FA, fatty acid; MUFA, monounsaturated fatty acid; PUFA, polyunsaturated fatty acid; SFA, saturated fatty acid.

The proportions of fatty acids in the epididymal and inguinal AT are presented in Figure 2. In the inguinal AT, Aa infection induced a significantly higher proportion of SFAs (Aa group: p = 0.027; Cpn + Aa group: p = 0.009; Figure 2A) and a lower proportion of PUFAs (Aa group: p = 0.006; Cpn + Aa group: p = 0.022; Figure 2C) compared to the control mice. The proportion of MUFAs was greater in the Cpn group (p = 0.018; Figure 2B).

In the epididymal AT, the proportions of SFAs were higher among all infected mice but the difference reached statistical significance only in the Aa group (p = 0.003; Figure 2A). The proportion of PUFAs was significantly lower in the Aa (p = 0.005) and Cpn + Aa groups (0.016) (Figure 2C). When the two AT depots were compared, the proportion of SFAs was lower in the epididymal AT than in the inguinal AT in the control group (17.1 ± 1.8 vs. 20.7 ± 2.1% of total FA, respectively; p = 0.002) and the Aa + Cpn group (19.0 ± 2.3 vs. 24.2 ± 3.0% of total FA, respectively; p = 0.001).

### Associations between serum and AT parameters

**Figure 3:**
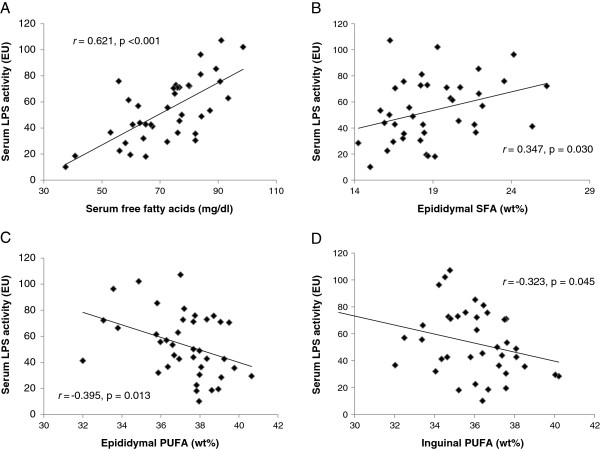
**Correlations between serum and AT parameters.** Bivariate correlations were determined using the Spearman test. **A)** Concentration of serum free fatty acids (mg/dl) and serum LPS activity (EU), **B)** serum LPS activity (EU) and epididymal SFAs (wt%), **C)** serum LPS activity (EU) and epididymal PUFAs (wt%), and **D)** serum LPS activity (EU) and inguinal PUFA s(wt%). EU, ELISA units; LPS, lipopolysaccharide; PUFA, polyunsaturated fatty acid; SFA, saturated fatty acid.

The associations between different serum and AT variables were determined by correlation analysis (data from all infection models and the controls combined) and presented as graphs in Figure 3. Serum LPS activity correlated positively with the serum FFA concentration (p < 0.001, Figure 3A) and with the proportion of SFA in the epididymal AT (p = 0.030, Figure 3B), and negatively with both inguinal (p = 0.045, Figure 3D) and epididymal (p = 0.013, Figure 3C) proportions of PUFA.

## Discussion

In the present study, we examined the inflammation- and lipid homeostasis-related effects of systemic *A. actinomycetemcomitans* and *C. pneumoniae* infections on AT derived from chow-fed apoE-deficient mice. These effects were examined on gene expression and fat composition level in both inguinal and epididymal AT depots. Recurrent Aa infection affected both AT depots significantly: inflammation-related biological pathways were enriched in inguinal AT and both inflammation- and lipid homeostasis-related pathways were enriched in epididymal AT. AT fat distribution was altered and SFAs were increased at the expense of PUFAs in both epididymal and inguinal AT among all *A. actinomycetemcomitans*-infected mice. The impact of chronic *C. pneumoniae* infection on AT depots was less prominent.

The mouse models used in the present study were designed to display chronic infections. Our Aa model is characterized by endotoxemia and systemic inflammation. The observed increased humoral immune responses can be somewhat expected, because we have previously shown that the serum levels of LPS activity, TNFα, and IgG antibodies against *A. actinomycetemcomitans* after 10 intravenous injections of live bacteria are significantly higher in the Aa group compared with the control group [19,20]. The persistence of *C. pneumonia* after three inoculations was also shown as the pathogen was recovered from the lungs of the mice [20]. Since atherosclerosis is a multiorgan disease, our aim was to broaden the study from the lipid depots in heart and liver to adipose tissue, which plays an important role in sustaining systemic inflammation and proatherogenic lipid profile.

Experimental acute endotoxemia has been shown to induce general insulin resistance and local and adaptive inflammation in human subcutaneous AT [27]. In our study, Aa infection enriched inflammation-related pathways in the inguinal AT. QPCR analysis verified up-regulated expression of *Mpo* and *Slpi*, and the most up-regulated genes in the microarray analysis were *S100a8* and *Ltf*. These innate immunity-associated gene products are considered anti-inflammatory mediators and they exhibit various antimicrobial functions such as promotion of oxidative activity responses [28-30]. Human Slpi and Ltf have been shown to co-localize in secondary granules and they are co-released after neutrophil activation [31]. Interestingly, increased plasma Slpi levels were associated with progressive metabolic dysfunction in men [32]. Despite the up-regulation in neutrophil activation-related genes, we did not observe changes in the mRNA expression of genes associated with classically or alternatively activated macrophages. The response of Aa infection in the epididymal AT was quite distinct. As in inguinal AT, the humoral antibacterial response and immunoglobulin-related pathways were enriched. However, the myeloid cell-specific signs of enhanced oxidative stress were absent, expression of *Slpi* was somewhat down-regulated and, instead, several lipid homeostasis-related biological pathways were enriched.

On AT fatty acid level, we observed a potentially adverse increase in the proportion of SFAs among all Aa-infected mice. Mengting et al. 2010 reported that compared with lean mice, obese mice have a higher level of SFAs in their epididymal AT [33]. An interesting study by Suganami et al. 2007 demonstrated that SFAs released by hypertrophied adipocytes activate the NF-κB pathway in the macrophages with ensuing TNF-α release [34]. The subsequent lipolytic release of SFAs creates a positive feed-back loop between adipocytes and macrophages. Importantly, the macrophage-derived TNF-α also resulted in NF-κB-dependent release of proinflammatory cytokines from the adipocytes. Both LPS and SFA have been shown to generate endoplasmic reticulum stress in primary human adipocytes [35]. Elevated levels of SFAs in both epididymal and inguinal AT depots in the Aa and Cpn + Aa groups suggested an increased proinflammatory potential of these AT depots. In addition, because PUFAs have been reported to inhibit the TLR dimerization induced by SFAs [36], the significantly decreased proportion of PUFAs among all *A. actinomycetemcomitans*-infected mice may intensify the effects of increased SFAs. The proportion of PUFAs also showed a significant inverse association with LPS serum activity. This result may be due to LPS-triggered enhanced oxidative stress in the AT, followed by the peroxidation of PUFAs. In addition to intracellular effects, LPS has been shown to elevate the levels of circulating FFAs in rodents *in vivo*[37]. This association was also found in our study as there was a strong positive correlation between serum LPS activity and the FFA level.

Lipid homeostasis-related biological pathways were enriched and four important *de novo* lipogenesis-related genes were moderately up-regulated in the epididymal AT in Aa group. The observed up-regulation of *Pcx* and *Fasn* could partially explain the increase in the SFA proportion. However, up-regulation of *de novo* lipogenesis genes was not observed in Cpn and Cpn + Aa groups, indicating the involvement of other regulatory pathways affecting SFA levels in the AT.

Chronic infection with *C. pneumoniae* did not enhance the pathways associated with humoral antibody response, and moreover, the effects on the AT depot fatty acid distribution were less evident than with Aa infection. However, the “Defense response” pathway which included four up-regulated defensins was enriched in the epididymal AT depot. Previously, *C. pneumoniae* infection *in vitro* was reported to induce human β-defensin 2 production mediated by the TLR4 pathway in monocytes [38]. Although both *C. pneumoniae* and *A. actinomycetemcomitans* are Gram-negative pathogens, the effects of Cpn infection differed considerably from those of Aa infection. This result may be due to the distinct infection routes and *C. pneumoniae* being an obligatory intracellular pathogen. In addition, the difference in the infection time points may also influence the outcome and, altogether, the character of chronic infection differs from the recurrent one. The combined infection model displayed a humoral antibody response and changes in AT depot FA distribution similar to those with single Aa infection. Nevertheless, indicators of the potentially amplified *de novo* lipogenesis in epididymal AT remained absent.

The major limitation of the study was the small number of mice available for the microarray analysis, which resulted in non-significant gene FC-results after FDR correction. Although the apoE-deficient mouse model has been utilized widely in atherosclerosis research, it possesses certain drawbacks, such as a somewhat non-physiological lipoprotein profile [39]. The adipocytes of apoE-deficient mice are smaller, contain fewer lipids, and accumulate less triglycerides from very low-density lipoproteins and chylomicrons compared with adipocytes derived from wild-type mice [40,41]. In addition, because apoE has been shown to protect against LPS-derived sepsis, apoE deficiency actually exposes mice to the deleterious and possibly proatherogenic effects of LPS [21]. Nevertheless, the augmented susceptibility to LPS may produce a more human-like response, because rodents are generally more resistant to LPS-mediated effects than humans. Our study design included only males and therefore we could only draw gender-specific conclusions. It is noteworthy that, in mice, approximately 20% of AT genes exhibit sexual dimorphism (FC >1.2), and the enriched functional categories with FC >3 include “Lipid metabolism” [42].

## Conclusions

The present study introduces *in vivo* evidence that systemic infection of *A. actinomycetemcomitans* activates inflammation-related biological pathways and alters the lipid homeostasis adversely in both inguinal and epididymal AT depots in apoE-deficient mice. The results suggest that repeated endotoxemia promotes AT dysfunction as shown by accumulation of saturated fat in the tissue and spill-over of free fatty acids into the circulation possibly via induced lipolysis. These observations may be useful when studying the potential mechanism linking adipose tissue to the proposed atherogenicity of the chronic infections.

## Abbreviations

Aa: *Aggregatibacter actinomycetemcomitans*; apoE: apolipoprotein E; AT: Adipose tissue; Cpn: *Chlamydia pneumoniae*; FC: Fold change; FDR: False discovery rate; FFA: Free fatty acid; GO: Gene ontology; IQR: Interquartile range; LPS: Lipopolysaccharide; MUFA: Monounsaturated fatty acid; PAI 1: Plasminogen activator inhibitor-1; PUFA: Polyunsaturated fatty acid; QPCR: Quantitative polymerase chain reaction; SD: Standard deviation; SFA: Saturated fatty acid; SPG: Sucrose-phosphate-glutamic acid buffer; TLR: Toll-like receptor; TNF-α: Tumour necrosis factor α.

## Competing interests

The authors declare that they have no competing interests.

## Authors’ contributions

KH, AMT, SL, MJ, PTK, ML, PS, GA, and PJP designed the study. KH performed experiments, analyzed and interpreted data, initially drafted the manuscript, and prepared tables and figures. AMT, SL, and IS performed experiments and edited the manuscript. MJ, PTK, ML, PS, GA, and PJP critically revised the manuscript and contributed to discussion. All authors read and approved the final manuscript.

## Supplementary Material

Additional file 1: Table S1 QPCR primer sequences. Click here for file

Additional file 2: Table S2 Differentially expressed genes in the inguinal AT transcriptome of recurrent *A. actinomycetemcomitans-*infected mice. Click here for file

Additional file 3: Table S3 Differentially expressed genes in the inguinal AT transcriptome of chronic *C. pneumoniae*-infected mice. Click here for file

Additional file 4: Table S4 Differentially expressed genes in the inguinal AT transcriptome of combined chronic *C. pneumoniae* and recurrent *A. actinomycetemcomitans*-infected mice. Click here for file

Additional file 5: Table S5 Differentially expressed genes in the epididymal AT transcriptome of recurrent *A. actinomycetemcomitans*-infected mice. Click here for file

Additional file 6: Table S6 Differentially expressed genes in the epididymal AT transcriptome of combined chronic *C. pneumoniae*-infected mice. Click here for file

Additional file 7: Table S7 Differentially expressed genes in the epididymal AT transcriptome of combined chronic *C. pneumoniae* and recurrent *A. actinomycetemcomitans*-infected mice. Click here for file
