# Supplementary material for: The effect of proatherogenic pathogens on adipose tissue transcriptome and fatty acid distribution in apolipoprotein E-deficient mice
Source: BMC Genomics. 2013 Oct 17;14:709. doi: 10.1186/1471-2164-14-709 (PMC4008135; doi:10.1186/1471-2164-14-709)
Supplement: Additional file 1: Table S1 — QPCR primer sequences. [file 1471-2164-14-709-S1.docx]

**Supplementary Table 1. QPCR primer sequences**

| **Target gene** | **Reference Sequence no.*** | **Forward primer 5’-3’** | **Reverse primer 5’-3’** |
| --- | --- | --- | --- |
| ***Cd68*** | NM 009853 | GCGGCTCCCTGTGTGTCTG | TTCTGTGGCTGTAGGTGTCATCG |
| ***Mrc-1*** | NM 008625 | GCTCGGGACTCTGGATTG | AGGCTCTGATGATGGACTTC |
| ***Mcp-1*** | NM 011333 | GAGCCAGACGGGAGGAAG | ATGGTGGTGGAGGAAGAGAG |
| ***Mif*** | NM 010798 | TCCACCTTCGCTTGAGTC | GGATAAACACAGAACACTACG |
| ***Slpi*** | NM 011414.3 | GCCCGGGAAAGCAGAGGTGC | TCCCAGGCTTCCTCCACACTGG |
| ***Mpo*** | NM 010824.2 | TTCTGGCAGGGGACATGCGCT | TGTGTGGCCAGCCGGTTATGC |
| ***Gapdh*** | NM 008084.2 | TTCAACGGCACAGTCAAGG | CTCCACGACATACTCAGCAC |

* National Center of Biotechnology Information (NCBI), <http://www.ncbi.nlm.nih.gov/>

The QPCR reaction primer concentration was 100 nM, except 200 nM for *Gapdh*.

AT, adipose tissue; *Gapdh*, glyceraldehyde-3-phosphate dehydrogenase; *Mcp-1*, monocyte chemoattractant protein 1; *Mif*, macrophage migration inhibitory factor, *Mrc-1*, mannose receptor C type 1; *Mpo*, myeloperoxidase; *Slpi*, secretory leukocyte peptidase inhibitor
