# Supplementary material for: The effect of proatherogenic pathogens on adipose tissue transcriptome and fatty acid distribution in apolipoprotein E-deficient mice
Source: BMC Genomics. 2013 Oct 17;14:709. doi: 10.1186/1471-2164-14-709 (PMC4008135; doi:10.1186/1471-2164-14-709)
Supplement: Additional file 2: Table S2 — Differentially expressed genes in the inguinal AT transcriptome of recurrent A. actinomycetemcomitans-infected mice. [file 1471-2164-14-709-S2.docx]

**Supplementary Table 2. Differentially expressed genes in the inguinal AT transcriptome of recurrent *A. actinomycetemcomitans-*infected mice**

| **Up-regulated genes^a^** | | | | **Down-regulated genes^a^** | | | |
| --- | --- | --- | --- | --- | --- | --- | --- |
| **Gene product** | **Fold change** | **P-value** | **Q-value^b^** | **Gene product** | **Fold change** | **P-value** | **Q-value^b^** |
| S100a8 | 13.27 | 0.013 | 0.417 | LOC623006 | 0.50 | 0.002 | 0.367 |
| S100a9 | 9.45 | 0.041 | 0.464 | Timp4 | 0.50 | 0.244 | 0.631 |
| Ltf | 6.62 | 0.078 | 0.509 | LOC380797 | 0.50 | 0.073 | 0.503 |
| LOC100048770 | 5.00 | 0.101 | 0.527 | Cmpk | 0.49 | 0.032 | 0.449 |
| IGKV8-31_AJ235957_Ig_kappa_variable_8-31_3 | 4.58 | 0.072 | 0.503 | Ndufb10 | 0.49 | 0.064 | 0.496 |
| Igkv13-84 | 4.24 | 0.156 | 0.572 | Hbb-b1 | 0.49 | 0.087 | 0.517 |
| IGKV2-137_AJ231263_Ig_kappa_variable_2-137_15 | 3.93 | 0.421 | 0.736 | LOC386270 | 0.49 | 0.029 | 0.446 |
| Igkv5-48 | 3.49 | 0.030 | 0.446 | Antxr1 | 0.49 | 0.171 | 0.580 |
| IGKV1-99_AJ231207_Ig_kappa_variable_1-99_1 | 3.37 | 0.574 | 0.819 | LOC382646 | 0.48 | 0.094 | 0.522 |
| Igl | 3.32 | 0.036 | 0.455 | Hsd3b2 | 0.48 | 0.022 | 0.440 |
| LOC630337 | 3.32 | 0.155 | 0.572 | Npr3 | 0.48 | 0.093 | 0.522 |
| LOC100046496 | 3.31 | 0.285 | 0.658 | A530055J02Rik | 0.47 | 0.042 | 0.465 |
| IGKV12-98_AJ235949_Ig_kappa_variable_12-98_12 | 3.31 | 0.298 | 0.665 | Arhgef1 | 0.47 | 0.097 | 0.525 |
| Prg2 | 3.25 | 0.030 | 0.446 | LOC333751 | 0.47 | 0.014 | 0.419 |
| Ighg | 3.25 | 0.034 | 0.449 | Cat | 0.47 | 0.065 | 0.496 |
| Ngp | 3.23 | 0.125 | 0.546 | Lep | 0.46 | 0.110 | 0.532 |
| LOC100047788 | 3.23 | 0.064 | 0.495 | Rps3a | 0.46 | 0.018 | 0.426 |
| IGHV1S119_L33961_Ig_heavy_variable_1S119_14 | 3.11 | 0.115 | 0.538 | LOC386005 | 0.46 | 0.026 | 0.441 |
| Igk-V5 | 3.06 | 0.009 | 0.401 | Ccdc6 | 0.46 | 0.020 | 0.433 |
| Igkv15-103 | 2.99 | 0.395 | 0.722 | 1200016E24Rik | 0.45 | 0.037 | 0.457 |
| D6Mit97 | 2.92 | 0.172 | 0.580 | Dcn | 0.45 | 0.061 | 0.493 |
| Igh-V11 | 2.91 | 0.089 | 0.519 | 8430408G22Rik | 0.45 | 0.270 | 0.647 |
| Igkv19-93 | 2.82 | 0.314 | 0.674 | Rhob | 0.45 | 0.033 | 0.449 |
| Igkv12-46 | 2.77 | 0.149 | 0.565 | 1110059G02Rik | 0.45 | 0.058 | 0.486 |
| Cox6a2 | 2.77 | 0.194 | 0.598 | Trappc6b | 0.45 | 0.041 | 0.464 |
| Retnlg | 2.74 | 0.163 | 0.576 | LOC386082 | 0.45 | 0.000 | 0.367 |
| Spon2 | 2.72 | 0.065 | 0.496 | Ptplb | 0.44 | 0.072 | 0.503 |
| LOC636696 | 2.70 | 0.170 | 0.579 | Cidea | 0.44 | 0.355 | 0.697 |
| IGKV9-120_V00804$J00566_Ig_kappa_variable_9-120_12 | 2.61 | 0.521 | 0.793 | G0s2 | 0.43 | 0.132 | 0.549 |
| LOC384415 | 2.49 | 0.186 | 0.592 | LOC668387 | 0.43 | 0.011 | 0.413 |
| LOC630347 | 2.42 | 0.254 | 0.637 | Aldh1a1 | 0.43 | 0.101 | 0.527 |
| Ighg3 | 2.36 | 0.246 | 0.632 | LOC277837 | 0.42 | 0.013 | 0.418 |
| IGHV1S30_X02462_Ig_heavy_variable_1S30_12 | 2.33 | 0.075 | 0.505 | LOC381365 | 0.42 | 0.005 | 0.396 |
| LOC232067 | 2.32 | 0.805 | 0.925 | LOC268700 | 0.42 | 0.009 | 0.401 |
| Chi3l3 | 2.30 | 0.160 | 0.574 | Siat7b | 0.42 | 0.003 | 0.382 |
| IGHV1S36_M13788_Ig_heavy_variable_1S36_40 | 2.25 | 0.173 | 0.582 | LOC386218 | 0.42 | 0.030 | 0.446 |
| Des | 2.19 | 0.091 | 0.519 | Ddr2 | 0.40 | 0.125 | 0.546 |
| LOC243431 | 2.16 | 0.503 | 0.782 | Map1lc3b | 0.40 | 0.006 | 0.397 |
| Cd177 | 2.12 | 0.169 | 0.579 | LOC386246 | 0.40 | 0.006 | 0.397 |
| LOC640696 | 2.09 | 0.259 | 0.641 | Acta1 | 0.39 | 0.814 | 0.928 |
| LOC232065 | 2.02 | 0.397 | 0.722 | LOC385825 | 0.39 | 0.047 | 0.473 |
| 1600002K03Rik | 2.01 | 0.081 | 0.511 | LOC665281 | 0.38 | 0.015 | 0.419 |
|  |  |  |  | Nr1d2 | 0.38 | 0.012 | 0.416 |
|  |  |  |  | Ywhag | 0.38 | 0.007 | 0.397 |
|  |  |  |  | Zbtb7a | 0.37 | 0.077 | 0.508 |
|  |  |  |  | LOC668038 | 0.36 | 0.007 | 0.397 |
|  |  |  |  | LOC665235 | 0.36 | 0.005 | 0.396 |
|  |  |  |  | LOC100042270 | 0.28 | 0.324 | 0.680 |
|  |  |  |  | LOC383196 | 0.25 | 0.287 | 0.659 |

^a^ Compared to the control group. Fold change limit 2.0.

^b^ Q-values are P-values corrected for multiple hypotheses using Benjamini-Hochberg false discovery rate.
