# Supplementary material for: The effect of proatherogenic pathogens on adipose tissue transcriptome and fatty acid distribution in apolipoprotein E-deficient mice
Source: BMC Genomics. 2013 Oct 17;14:709. doi: 10.1186/1471-2164-14-709 (PMC4008135; doi:10.1186/1471-2164-14-709)
Supplement: Additional file 3: Table S3 — Differentially expressed genes in the inguinal AT transcriptome of chronic C. pneumoniae-infected mice. [file 1471-2164-14-709-S3.docx]

**Supplementary Table 3. Differentially expressed genes in the inguinal AT transcriptome of chronic *C. pneumoniae*-infected mice**

| **Up-regulated genes^a^** | | | | **Down-regulated genes^a^** | | | |
| --- | --- | --- | --- | --- | --- | --- | --- |
| **Gene product** | **Fold change** | **P-value** | **Q-value^b^** | **Gene product** | **Fold change** | **P-value** | **Q-value^b^** |
| IGKV1-99_AJ231207_Ig_kappa_variable_1-99_1 | 3.89 | 0.818 | 0.926 | Gpx8 | 0.50 | 0.036 | 0.376 |
| Tnnc2 | 3.65 | 0.495 | 0.756 | LOC665281 | 0.50 | 0.002 | 0.306 |
| Usp18 | 2.57 | 0.026 | 0.362 | LOC386246 | 0.50 | 0.005 | 0.306 |
| Ifit3 | 2.37 | 0.030 | 0.365 | LOC271505 | 0.49 | 0.306 | 0.633 |
| A530020H22Rik | 2.33 | 0.003 | 0.306 | LOC641178 | 0.49 | 0.082 | 0.432 |
| LOC100046552 | 2.20 | 0.869 | 0.949 | Hbb-b1 | 0.48 | 0.063 | 0.408 |
| Fcrls | 2.13 | 0.014 | 0.334 | Nat8l | 0.48 | 0.457 | 0.733 |
| LOC384411 | 2.13 | 0.208 | 0.557 | 8430408G22Rik | 0.48 | 0.009 | 0.312 |
| Osr2 | 2.09 | 0.027 | 0.362 | Nrg4 | 0.48 | 0.142 | 0.496 |
| Gpc3 | 2.08 | 0.006 | 0.306 | IGHV1S28_X02460_Ig_heavy_variable_1S28_13 | 0.48 | 0.010 | 0.320 |
| C230075M21Rik | 2.04 | 0.025 | 0.362 | LOC386005 | 0.47 | 0.008 | 0.311 |
| Ppap2b | 2.02 | 0.002 | 0.306 | Ywhag | 0.47 | 0.026 | 0.362 |
| Slfn1 | 2.02 | 0.052 | 0.397 | Actr3 | 0.46 | 0.001 | 0.302 |
| Eif2ak2 | 2.02 | 0.021 | 0.352 | LOC545472 | 0.46 | 0.048 | 0.394 |
|  |  |  |  | LOC226017 | 0.46 | 0.051 | 0.395 |
|  |  |  |  | Rps3a | 0.45 | 0.016 | 0.343 |
|  |  |  |  | LOC674147 | 0.45 | 0.003 | 0.306 |
|  |  |  |  | Nr1d2 | 0.44 | 0.795 | 0.914 |
|  |  |  |  | Elovl6 | 0.44 | 0.109 | 0.465 |
|  |  |  |  | LOC213684 | 0.44 | 0.114 | 0.470 |
|  |  |  |  | IGHV12S1_M22439_Ig_heavy_variable_12S1_339 | 0.44 | 0.041 | 0.384 |
|  |  |  |  | LOC668387 | 0.44 | 0.009 | 0.313 |
|  |  |  |  | LOC380707 | 0.43 | 0.354 | 0.668 |
|  |  |  |  | LOC100047788 | 0.43 | 0.356 | 0.669 |
|  |  |  |  | Mod1 | 0.43 | 0.049 | 0.394 |
|  |  |  |  | Ddit4 | 0.43 | 0.009 | 0.312 |
|  |  |  |  | LOC674707 | 0.42 | 0.007 | 0.306 |
|  |  |  |  | Acaa2 | 0.41 | 0.247 | 0.585 |
|  |  |  |  | LOC385291 | 0.40 | 0.004 | 0.306 |
|  |  |  |  | LOC381365 | 0.40 | 0.004 | 0.306 |
|  |  |  |  | LOC665235 | 0.40 | 0.054 | 0.397 |
|  |  |  |  | LOC672342 | 0.36 | 0.405 | 0.702 |
|  |  |  |  | LOC672339 | 0.32 | 0.474 | 0.744 |
|  |  |  |  | LOC668038 | 0.26 | 0.107 | 0.463 |
|  |  |  |  | LOC100042270 | 0.25 | 0.105 | 0.461 |
|  |  |  |  | LOC383196 | 0.21 | 0.423 | 0.712 |
|  |  |  |  | Ighg3 | 0.17 | 0.309 | 0.635 |

^a^ Compared to the control group. Fold change limit 2.0.

^b^ Q-values are P-values corrected for multiple hypotheses using Benjamini-Hochberg false discovery rate.
