# Supplementary material for: The effect of proatherogenic pathogens on adipose tissue transcriptome and fatty acid distribution in apolipoprotein E-deficient mice
Source: BMC Genomics. 2013 Oct 17;14:709. doi: 10.1186/1471-2164-14-709 (PMC4008135; doi:10.1186/1471-2164-14-709)
Supplement: Additional file 4: Table S4 — Differentially expressed genes in the inguinal AT transcriptome of combined chronic C. pneumoniae and recurrent A. actinomycetemcomitans-infected mice. [file 1471-2164-14-709-S4.docx]

**Supplementary Table 4. Differentially expressed genes in the inguinal AT transcriptome of combined chronic *C. pneumoniae* and recurrent *A. actinomycetemcomitans*-infected mice**

| **Up-regulated genes^a^** | | | | **Down-regulated genes^a^** | | | | |
| --- | --- | --- | --- | --- | --- | --- | --- | --- |
| **Gene product** | **Fold change** | **P-value** | **Q-value^b^** | | **Gene product** | **Fold change** | **P-value** | **Q-value^b^** |
| Igkv15-103 | 19.20 | 0.119 | 0.993 | | Dpt | 0.49 | 0.169 | 0.993 |
| S100a9 | 11.29 | 0.057 | 0.993 | | Ddx17 | 0.49 | 0.144 | 0.993 |
| S100a8 | 10.96 | 0.034 | 0.993 | | Lep | 0.46 | 0.386 | 0.993 |
| IGHV1S119_L33961_Ig_heavy_variable_1S119_14 | 8.08 | 0.062 | 0.993 | | Bmp3 | 0.46 | 0.306 | 0.993 |
| IGKV12-98_AJ235949_Ig_kappa_variable_12-98_12 | 8.05 | 0.182 | 0.993 | | Tnnc2 | 0.46 | 0.610 | 0.993 |
| LOC100047788 | 7.81 | 0.038 | 0.993 | | Igfbp6 | 0.46 | 0.158 | 0.993 |
| LOC640696 | 7.60 | 0.088 | 0.993 | | Adh7 | 0.43 | 0.137 | 0.993 |
| IGKV8-31_AJ235957_Ig_kappa_variable_8-31_3 | 7.52 | 0.079 | 0.993 | | Aldh1a1 | 0.43 | 0.114 | 0.993 |
| LOC232065 | 5.84 | 0.089 | 0.993 | | Sncg | 0.40 | 0.214 | 0.993 |
| Igk-V5 | 5.76 | 0.098 | 0.993 | | Slc6a13 | 0.37 | 0.176 | 0.993 |
| IGHV1S28_X02460_Ig_heavy_variable_1S28_13 | 5.44 | 0.013 | 0.993 | | Sncg | 0.36 | 0.164 | 0.993 |
| Ltf | 4.73 | 0.087 | 0.993 | | Acta1 | 0.34 | 0.539 | 0.993 |
| LOC637260 | 4.32 | 0.003 | 0.993 | |  |  |  |  |
| Igh-4 | 4.16 | 0.091 | 0.993 | |  |  |  |  |
| IGHV1S59_L17134_Ig_heavy_variable_1S59_150 | 4.03 | 0.258 | 0.993 | |  |  |  |  |
| Ighg | 3.90 | 0.048 | 0.993 | |  |  |  |  |
| LOC232067 | 3.88 | 0.295 | 0.993 | |  |  |  |  |
| IGHV1S34_X02467_Ig_heavy_variable_1S34_71 | 3.87 | 0.183 | 0.993 | |  |  |  |  |
| LOC100048770 | 3.85 | 0.057 | 0.993 | |  |  |  |  |
| IGKV9-120_V00804$J00566_Ig_kappa_variable_9-120_12 | 3.76 | 0.595 | 0.993 | |  |  |  |  |
| IGHV1S30_X02462_Ig_heavy_variable_1S30_12 | 3.72 | 0.061 | 0.993 | |  |  |  |  |
| Gm16971 | 3.56 | 0.173 | 0.993 | |  |  |  |  |
| IGHG1_J00453$V00793_Ig_heavy_constant_gamma_1_792 | 3.46 | 0.113 | 0.993 | |  |  |  |  |
| LOC434031 | 3.31 | 0.298 | 0.993 | |  |  |  |  |
| Ngp | 3.29 | 0.092 | 0.993 | |  |  |  |  |
| LOC384415 | 3.15 | 0.256 | 0.993 | |  |  |  |  |
| Prg2 | 3.04 | 0.122 | 0.993 | |  |  |  |  |
| LOC674147 | 3.04 | 0.018 | 0.993 | |  |  |  |  |
| Igkv5-48 | 3.02 | 0.254 | 0.993 | |  |  |  |  |
| LOC626347 | 2.93 | 0.056 | 0.993 | |  |  |  |  |
| LOC272683 | 2.91 | 0.140 | 0.993 | |  |  |  |  |
| D6Mit97 | 2.89 | 0.311 | 0.993 | |  |  |  |  |
| Igl-V1 | 2.84 | 0.256 | 0.993 | |  |  |  |  |
| Igl | 2.28 | 0.063 | 0.993 | |  |  |  |  |
| Ighv1-62 | 2.21 | 0.183 | 0.993 | |  |  |  |  |
| IGHV1S14_K00707$X00161_Ig_heavy_variable_1S14_164 | 2.18 | 0.091 | 0.993 | |  |  |  |  |
| IGKV4-80_AJ231213_Ig_kappa_variable_4-80_91 | 2.17 | 0.049 | 0.993 | |  |  |  |  |
| LOC672329 | 2.13 | 0.085 | 0.993 | |  |  |  |  |
| IGHV1S120_AF025443_Ig_heavy_variable_1S120_8 | 2.12 | 0.192 | 0.993 | |  |  |  |  |
| LOC626583 | 2.12 | 0.825 | 0.996 | |  |  |  |  |
| IGHV1S35_M12376_Ig_heavy_variable_1S35_13 | 2.11 | 0.086 | 0.993 | |  |  |  |  |
| Ighg3 | 2.06 | 0.480 | 0.993 | |  |  |  |  |
| LOC630305 | 2.04 | 0.210 | 0.993 | |  |  |  |  |
| Chi3l3 | 2.03 | 0.179 | 0.993 | |  |  |  |  |
| Igkv12-89 | 2.01 | 0.122 | 0.993 | |  |  |  |  |

^a^ Compared to the control group. Fold change limit 2.0.

^b^ Q-values are P-values corrected for multiple hypotheses using Benjamini-Hochberg false discovery rate.
