# Supplementary material for: The effect of proatherogenic pathogens on adipose tissue transcriptome and fatty acid distribution in apolipoprotein E-deficient mice
Source: BMC Genomics. 2013 Oct 17;14:709. doi: 10.1186/1471-2164-14-709 (PMC4008135; doi:10.1186/1471-2164-14-709)
Supplement: Additional file 5: Table S5 — Differentially expressed genes in the epididymal AT transcriptome of recurrent A. actinomycetemcomitans-infected mice. [file 1471-2164-14-709-S5.docx]

**Supplementary Table 5. Differentially expressed genes in the epididymal AT transcriptome of recurrent *A. actinomycetemcomitans*-infected mice**

| **Up-regulated genes^a^** | | | | **Down-regulated genes^a^** | | | | |
| --- | --- | --- | --- | --- | --- | --- | --- | --- |
| **Gene product** | **Fold change** | **P-value** | **Q-value^b^** | | **Gene product** | **Fold change** | **P-value** | **Q-value^b^** |
| LOC100047788 | 22.35 | 0.018 | 0.409 | | Itga11 | 0.49 | 0.104 | 0.520 |
| Igl-V1 | 8.49 | 0.046 | 0.457 | | Gfpt2 | 0.47 | 0.079 | 0.496 |
| Ighg | 7.68 | 0.325 | 0.685 | | Chst4 | 0.46 | 0.096 | 0.514 |
| Ighg3 | 4.77 | 0.095 | 0.514 | | Lyz1 | 0.46 | 0.119 | 0.533 |
| Igk-C | 4.57 | 0.250 | 0.632 | | Bcl6 | 0.44 | 0.085 | 0.503 |
| Igk-V5 | 4.16 | 0.050 | 0.459 | | Erdr1 | 0.37 | 0.234 | 0.623 |
| LOC384413 | 3.45 | 0.046 | 0.457 | | Itga11 | 0.49 | 0.104 | 0.520 |
| LOC100047162 | 3.34 | 0.109 | 0.524 | | Gfpt2 | 0.47 | 0.079 | 0.496 |
| Elovl6 | 3.21 | 0.185 | 0.586 | | Chst4 | 0.46 | 0.096 | 0.514 |
| Igl-V1 | 2.92 | 0.281 | 0.654 | | Lyz1 | 0.46 | 0.119 | 0.533 |
| Pcx | 2.82 | 0.386 | 0.723 | | Bcl6 | 0.44 | 0.085 | 0.503 |
| IGKV3-2_X16954_Ig_kappa_variable_3-2_18 | 2.80 | 0.021 | 0.410 | | Erdr1 | 0.37 | 0.234 | 0.623 |
| LOC207685 | 2.72 | 0.490 | 0.780 | |  |  |  |  |
| Fabp4 | 2.60 | 0.218 | 0.613 | |  |  |  |  |
| Cidea | 2.53 | 0.118 | 0.532 | |  |  |  |  |
| LOC637227 | 2.51 | 0.016 | 0.409 | |  |  |  |  |
| LOC669053 | 2.50 | 0.275 | 0.650 | |  |  |  |  |
| LOC676136 | 2.49 | 0.211 | 0.607 | |  |  |  |  |
| A530020H22Rik | 2.47 | 0.223 | 0.616 | |  |  |  |  |
| Mod1 | 2.28 | 0.054 | 0.469 | |  |  |  |  |
| Thrsp | 2.26 | 0.001 | 0.381 | |  |  |  |  |
| LOC245892 | 2.25 | 0.018 | 0.409 | |  |  |  |  |
| Klf6 | 2.24 | 0.012 | 0.399 | |  |  |  |  |
| Scd2 | 2.15 | 0.012 | 0.401 | |  |  |  |  |
| 9130213B05Rik | 2.12 | 0.002 | 0.381 | |  |  |  |  |
| Slc15a2 | 2.08 | 0.025 | 0.417 | |  |  |  |  |
| Dusp4 | 2.06 | 0.061 | 0.476 | |  |  |  |  |
| Lrtm1 | 2.02 | 0.069 | 0.484 | |  |  |  |  |
| IGLC2_J00595_Ig_lambda_constant_2_14 | 2.01 | 0.072 | 0.487 | |  |  |  |  |
| Insig1 | 2.01 | 0.274 | 0.649 | |  |  |  |  |

|  |
| --- |

^a^ Compared to the control group. Fold change limit 2.0.

^b^ Q-values are P-values corrected for multiple hypotheses using Benjamini-Hochberg false discovery rate.
