# Supplementary material for: The effect of proatherogenic pathogens on adipose tissue transcriptome and fatty acid distribution in apolipoprotein E-deficient mice
Source: BMC Genomics. 2013 Oct 17;14:709. doi: 10.1186/1471-2164-14-709 (PMC4008135; doi:10.1186/1471-2164-14-709)
Supplement: Additional file 6: Table S6 — Differentially expressed genes in the epididymal AT transcriptome of combined chronic C. pneumoniae-infected mice. [file 1471-2164-14-709-S6.docx]

**Supplementary Table 6. Differentially expressed genes in the epididymal AT transcriptome of combined chronic *C. pneumoniae*-infected mice**

| **Up-regulated genes^a^** | | | | **Down-regulated genes^a^** | | | | |
| --- | --- | --- | --- | --- | --- | --- | --- | --- |
| **Gene product** | **Fold change** | **P-value** | **Q-value^b^** | | **Gene product** | **Fold change** | **P-value** | **Q-value^b^** |
| Cuzd1 | 28.63 | 0.172 | 0.582 | | Thrsp | 0.49 | 0.316 | 0.665 |
| Prm1 | 24.70 | 0.108 | 0.545 | | Ccl5 | 0.48 | 0.245 | 0.620 |
| BC038167 | 13.74 | 0.183 | 0.587 | | Ffar3 | 0.48 | 0.005 | 0.451 |
| Iyd | 10.43 | 0.179 | 0.586 | | Tppp | 0.48 | 0.037 | 0.495 |
| Dynlrb2 | 10.20 | 0.203 | 0.597 | | Hist1h3d | 0.47 | 0.162 | 0.578 |
| Cldn2 | 9.43 | 0.178 | 0.585 | | Gpd1 | 0.47 | 0.074 | 0.525 |
| Pdzk1 | 9.10 | 0.162 | 0.578 | | Paqr9 | 0.46 | 0.180 | 0.586 |
| Slc30a3 | 7.75 | 0.190 | 0.592 | | Hist2h2ac | 0.46 | 0.134 | 0.563 |
| Mmd2 | 7.71 | 0.137 | 0.565 | | Hist1h4k | 0.46 | 0.147 | 0.569 |
| Mia1 | 7.43 | 0.172 | 0.582 | | 1110059M19Rik | 0.46 | 0.151 | 0.571 |
| Clic6 | 7.19 | 0.110 | 0.546 | | Bmp3 | 0.44 | 0.032 | 0.489 |
| Mt3 | 6.85 | 0.173 | 0.583 | | Hist1h4i | 0.43 | 0.161 | 0.578 |
| Kcnk1 | 6.41 | 0.187 | 0.590 | | Tnfrsf11b | 0.43 | 0.086 | 0.532 |
| Lrp2 | 5.70 | 0.204 | 0.598 | | Nat8l | 0.43 | 0.306 | 0.659 |
| Alox12 | 5.61 | 0.164 | 0.579 | | S100a8 | 0.42 | 0.305 | 0.658 |
| LOC100048331 | 5.17 | 0.180 | 0.586 | | Hist1h4j | 0.41 | 0.154 | 0.573 |
| 1600029I14Rik | 5.15 | 0.189 | 0.591 | | Mod1 | 0.41 | 0.340 | 0.682 |
| Acsbg1 | 5.04 | 0.153 | 0.573 | | Ccl11 | 0.39 | 0.032 | 0.489 |
| Sox9 | 4.94 | 0.187 | 0.590 | | C130008L17Rik | 0.38 | 0.179 | 0.586 |
| Bbox1 | 4.85 | 0.175 | 0.583 | | Orm1 | 0.34 | 0.156 | 0.574 |
| Nme7 | 4.57 | 0.190 | 0.592 | | Orm2 | 0.33 | 0.046 | 0.501 |
| Cldn11 | 4.46 | 0.151 | 0.571 | | Cox8b | 0.31 | 0.034 | 0.493 |
| 2010001J22Rik | 4.45 | 0.157 | 0.575 | | Acta1 | 0.15 | 0.064 | 0.516 |
| Sectm1b | 4.44 | 0.193 | 0.592 | |  |  |  |  |
| Mmrn1 | 4.30 | 0.167 | 0.580 | |  |  |  |  |
| Fbp2 | 4.29 | 0.143 | 0.568 | |  |  |  |  |
| 1110049B09Rik | 4.24 | 0.185 | 0.589 | |  |  |  |  |
| Cldn10 | 4.06 | 0.151 | 0.571 | |  |  |  |  |
| C7 | 4.04 | 0.157 | 0.575 | |  |  |  |  |
| Pcp4l1 | 4.01 | 0.141 | 0.567 | |  |  |  |  |
| Iyd | 3.81 | 0.206 | 0.598 | |  |  |  |  |
| Acsbg1 | 3.80 | 0.180 | 0.586 | |  |  |  |  |
| Atp1b1 | 3.75 | 0.280 | 0.643 | |  |  |  |  |
| EG665033 | 3.67 | 0.166 | 0.580 | |  |  |  |  |
| Tnp1 | 3.64 | 0.180 | 0.586 | |  |  |  |  |
| Ddit4l | 3.62 | 0.150 | 0.571 | |  |  |  |  |
| Slc27a2 | 3.49 | 0.193 | 0.593 | |  |  |  |  |
| Dnali1 | 3.49 | 0.155 | 0.574 | |  |  |  |  |
| Ighg | 3.48 | 0.111 | 0.546 | |  |  |  |  |
| 1700024G13Rik | 3.47 | 0.149 | 0.570 | |  |  |  |  |
| A530020H22Rik | 3.43 | 0.040 | 0.495 | |  |  |  |  |
| Odf1 | 3.43 | 0.179 | 0.586 | |  |  |  |  |
| LOC384413 | 3.42 | 0.135 | 0.563 | |  |  |  |  |
| Krt8 | 3.42 | 0.282 | 0.645 | |  |  |  |  |
| Bex4 | 3.41 | 0.173 | 0.583 | |  |  |  |  |
| Ldhc | 3.41 | 0.197 | 0.594 | |  |  |  |  |
| Ppil6 | 3.40 | 0.149 | 0.570 | |  |  |  |  |
| Nt5e | 3.34 | 0.112 | 0.547 | |  |  |  |  |
| Acpp | 3.33 | 0.117 | 0.549 | |  |  |  |  |
| BC038167 | 3.31 | 0.212 | 0.600 | |  |  |  |  |
| Bex2 | 3.28 | 0.179 | 0.586 | |  |  |  |  |
| Pacrg | 3.27 | 0.175 | 0.583 | |  |  |  |  |
| Napsa | 3.26 | 0.153 | 0.573 | |  |  |  |  |
| Oaz3 | 3.24 | 0.154 | 0.573 | |  |  |  |  |
| Igk-C | 3.24 | 0.033 | 0.489 | |  |  |  |  |
| 1500015O10Rik | 3.23 | 0.186 | 0.590 | |  |  |  |  |
| Nme7 | 3.22 | 0.241 | 0.617 | |  |  |  |  |
| 9030624O13Rik | 3.19 | 0.158 | 0.576 | |  |  |  |  |
| Mycbpap | 3.18 | 0.165 | 0.579 | |  |  |  |  |
| A530026G17 | 3.18 | 0.039 | 0.495 | |  |  |  |  |
| LOC100047628 | 3.16 | 0.030 | 0.489 | |  |  |  |  |
| Calml3 | 3.10 | 0.223 | 0.606 | |  |  |  |  |
| LOC381284 | 3.10 | 0.190 | 0.592 | |  |  |  |  |
| Atad4 | 3.07 | 0.211 | 0.600 | |  |  |  |  |
| Smcp | 3.06 | 0.159 | 0.577 | |  |  |  |  |
| 1700016K19Rik | 3.05 | 0.172 | 0.583 | |  |  |  |  |
| B4galnt2 | 3.03 | 0.215 | 0.602 | |  |  |  |  |
| Mns1 | 3.01 | 0.159 | 0.577 | |  |  |  |  |
| Cfi | 2.97 | 0.189 | 0.591 | |  |  |  |  |
| Elf3 | 2.97 | 0.189 | 0.591 | |  |  |  |  |
| 4931407G18Rik | 2.94 | 0.140 | 0.567 | |  |  |  |  |
| Gja1 | 2.92 | 0.170 | 0.582 | |  |  |  |  |
| Gcap27 | 2.91 | 0.207 | 0.598 | |  |  |  |  |
| Sh3gl2 | 2.88 | 0.150 | 0.571 | |  |  |  |  |
| OTTMUSG00000015852 | 2.88 | 0.276 | 0.641 | |  |  |  |  |
| Spag6 | 2.86 | 0.186 | 0.590 | |  |  |  |  |
| Igsf11 | 2.85 | 0.219 | 0.604 | |  |  |  |  |
| Inmt | 2.84 | 0.187 | 0.590 | |  |  |  |  |
| Kcnk1 | 2.82 | 0.215 | 0.602 | |  |  |  |  |
| LOC100048554 | 2.81 | 0.349 | 0.687 | |  |  |  |  |
| Sult1c2 | 2.81 | 0.136 | 0.564 | |  |  |  |  |
| Sfrp1 | 2.80 | 0.190 | 0.592 | |  |  |  |  |
| Napsa | 2.79 | 0.181 | 0.587 | |  |  |  |  |
| Defb29 | 2.73 | 0.276 | 0.642 | |  |  |  |  |
| Gpnmb | 2.70 | 0.083 | 0.529 | |  |  |  |  |
| Slc27a2 | 2.69 | 0.191 | 0.592 | |  |  |  |  |
| Fxyd6 | 2.69 | 0.241 | 0.617 | |  |  |  |  |
| C030048H21Rik | 2.67 | 0.226 | 0.608 | |  |  |  |  |
| 4932425I24Rik | 2.66 | 0.206 | 0.598 | |  |  |  |  |
| Dsg2 | 2.66 | 0.254 | 0.626 | |  |  |  |  |
| Wwc1 | 2.66 | 0.239 | 0.616 | |  |  |  |  |
| D630003M21Rik | 2.66 | 0.183 | 0.588 | |  |  |  |  |
| Ccdc108 | 2.65 | 0.121 | 0.551 | |  |  |  |  |
| Sp5 | 2.64 | 0.146 | 0.569 | |  |  |  |  |
| Ttc39a | 2.60 | 0.193 | 0.592 | |  |  |  |  |
| Kcnk5 | 2.58 | 0.172 | 0.582 | |  |  |  |  |
| Lrrc48 | 2.57 | 0.162 | 0.578 | |  |  |  |  |
| Lrrc34 | 2.56 | 0.120 | 0.551 | |  |  |  |  |
| Srd5a2 | 2.56 | 0.160 | 0.578 | |  |  |  |  |
| Nme7 | 2.55 | 0.203 | 0.597 | |  |  |  |  |
| Bex2 | 2.54 | 0.146 | 0.569 | |  |  |  |  |
| Slc15a2 | 2.53 | 0.038 | 0.495 | |  |  |  |  |
| 9930105H17Rik | 2.52 | 0.132 | 0.562 | |  |  |  |  |
| 2010001J22Rik | 2.51 | 0.141 | 0.567 | |  |  |  |  |
| BC021891 | 2.50 | 0.148 | 0.570 | |  |  |  |  |
| Ccdc113 | 2.49 | 0.194 | 0.593 | |  |  |  |  |
| Ldhc | 2.44 | 0.161 | 0.578 | |  |  |  |  |
| Slc22a1 | 2.43 | 0.135 | 0.563 | |  |  |  |  |
| 1700007G11Rik | 2.43 | 0.077 | 0.528 | |  |  |  |  |
| Cyb561 | 2.43 | 0.238 | 0.616 | |  |  |  |  |
| 9230002F21Rik | 2.39 | 0.285 | 0.647 | |  |  |  |  |
| 1110049B09Rik | 2.33 | 0.158 | 0.576 | |  |  |  |  |
| Cda | 2.32 | 0.146 | 0.569 | |  |  |  |  |
| Capsl | 2.31 | 0.161 | 0.578 | |  |  |  |  |
| Pdzk1 | 2.30 | 0.161 | 0.578 | |  |  |  |  |
| Igh-VJ558 | 2.29 | 0.070 | 0.522 | |  |  |  |  |
| OTTMUSG00000017677 | 2.29 | 0.190 | 0.592 | |  |  |  |  |
| scl0002540.1_6 | 2.28 | 0.175 | 0.583 | |  |  |  |  |
| 4933434I06Rik | 2.27 | 0.127 | 0.558 | |  |  |  |  |
| Col8a2 | 2.27 | 0.193 | 0.593 | |  |  |  |  |
| Nalp6 | 2.26 | 0.151 | 0.571 | |  |  |  |  |
| Chad | 2.26 | 0.130 | 0.560 | |  |  |  |  |
| Tm4sf4 | 2.26 | 0.155 | 0.574 | |  |  |  |  |
| LOC380653 | 2.24 | 0.246 | 0.620 | |  |  |  |  |
| Ccdc19 | 2.24 | 0.110 | 0.546 | |  |  |  |  |
| Krt18 | 2.23 | 0.290 | 0.650 | |  |  |  |  |
| Vil1 | 2.23 | 0.139 | 0.566 | |  |  |  |  |
| Avpr1a | 2.23 | 0.043 | 0.496 | |  |  |  |  |
| Inmt | 2.19 | 0.220 | 0.605 | |  |  |  |  |
| Lrrc50 | 2.19 | 0.151 | 0.571 | |  |  |  |  |
| Cx3cl1 | 2.17 | 0.123 | 0.553 | |  |  |  |  |
| Igsf11 | 2.16 | 0.188 | 0.591 | |  |  |  |  |
| Ddx26 | 2.15 | 0.174 | 0.583 | |  |  |  |  |
| Defb38 | 2.15 | 0.306 | 0.659 | |  |  |  |  |
| Gja4 | 2.13 | 0.066 | 0.518 | |  |  |  |  |
| Cyp2d9 | 2.12 | 0.158 | 0.576 | |  |  |  |  |
| Cds1 | 2.10 | 0.194 | 0.593 | |  |  |  |  |
| Gyk | 2.10 | 0.145 | 0.569 | |  |  |  |  |
| Klhdc9 | 2.09 | 0.101 | 0.540 | |  |  |  |  |
| Ramp1 | 2.09 | 0.193 | 0.592 | |  |  |  |  |
| Defb36 | 2.08 | 0.189 | 0.591 | |  |  |  |  |
| A830087P12Rik | 2.08 | 0.146 | 0.569 | |  |  |  |  |
| Odf2 | 2.07 | 0.137 | 0.565 | |  |  |  |  |
| Defb29 | 2.07 | 0.180 | 0.586 | |  |  |  |  |
| Slc39a4 | 2.06 | 0.156 | 0.574 | |  |  |  |  |
| LOC675572 | 2.06 | 0.309 | 0.660 | |  |  |  |  |
| Gchfr | 2.03 | 0.128 | 0.559 | |  |  |  |  |
| LOC331139 | 2.03 | 0.106 | 0.544 | |  |  |  |  |
| Arg2 | 2.02 | 0.196 | 0.593 | |  |  |  |  |
| Tcea3 | 2.00 | 0.163 | 0.579 | |  |  |  |  |
| Lrrn1 | 2.00 | 0.033 | 0.489 | |  |  |  |  |

|  |
| --- |

^a^ Compared to the control group. Fold change limit 2.0.

^b^ Q-values are P-values corrected for multiple hypotheses using Benjamini-Hochberg false discovery rate.
