# Supplementary material for: The effect of proatherogenic pathogens on adipose tissue transcriptome and fatty acid distribution in apolipoprotein E-deficient mice
Source: BMC Genomics. 2013 Oct 17;14:709. doi: 10.1186/1471-2164-14-709 (PMC4008135; doi:10.1186/1471-2164-14-709)
Supplement: Additional file 7: Table S7 — Differentially expressed genes in the epididymal AT transcriptome of combined chronic C. pneumoniae and recurrent A. actinomycetemcomitans-infected mice. [file 1471-2164-14-709-S7.docx]

**Supplementary Table 7. Differentially expressed genes in the epididymal AT transcriptome of combined chronic *C. pneumoniae* and recurrent *A. actinomycetemcomitans*-infected mice**

| **Up-regulated genes^a^** | | | | **Down-regulated genes^a^** | | | | |
| --- | --- | --- | --- | --- | --- | --- | --- | --- |
| **Gene product** | **Fold change** | **P-value** | **Q-value^b^** | | **Gene product** | **Fold change** | **P-value** | **Q-value^b^** |
| LOC100047788 | 37.73 | 0.006 | 0.420 | | Hist1h3d | 0.49 | 0.022 | 0.447 |
| Ighg | 25.44 | 0.000 | 0.316 | | 1110059M19Rik | 0.49 | 0.081 | 0.509 |
| Cuzd1 | 17.34 | 0.171 | 0.583 | | Sfrp5 | 0.48 | 0.029 | 0.460 |
| Igk-V5 | 14.15 | 0.002 | 0.408 | | Hist1h4h | 0.47 | 0.019 | 0.445 |
| Ighg3 | 11.24 | 0.233 | 0.621 | | BC064033 | 0.47 | 0.182 | 0.589 |
| Igl-V1 | 9.08 | 0.161 | 0.576 | | Trp53inp2 | 0.47 | 0.044 | 0.475 |
| Igk-C | 8.13 | 0.007 | 0.420 | | Upk1b | 0.46 | 0.107 | 0.531 |
| Iyd | 7.00 | 0.187 | 0.591 | | scl0002507.1_236 | 0.45 | 0.090 | 0.516 |
| BC038167 | 6.85 | 0.206 | 0.604 | | Hist2h2ac | 0.45 | 0.007 | 0.420 |
| Cldn2 | 6.28 | 0.179 | 0.588 | | C130008L17Rik | 0.45 | 0.062 | 0.489 |
| Igkv12-46 | 4.85 | 0.038 | 0.467 | | 1200016E24Rik | 0.44 | 0.026 | 0.455 |
| Dynlrb2 | 4.69 | 0.201 | 0.600 | | Hist1h4j | 0.43 | 0.016 | 0.443 |
| Pdzk1 | 4.66 | 0.154 | 0.571 | | Itga11 | 0.41 | 0.052 | 0.483 |
| Slc30a3 | 4.63 | 0.193 | 0.596 | | Hist1h4k | 0.41 | 0.007 | 0.420 |
| Mt3 | 4.25 | 0.169 | 0.582 | | Hist1h4i | 0.41 | 0.003 | 0.408 |
| IGKV3-2_X16954_Ig_kappa_variable_3-2_18 | 4.16 | 0.312 | 0.677 | | Upk3b | 0.41 | 0.129 | 0.549 |
| LOC637227 | 4.10 | 0.107 | 0.532 | | Lyz1 | 0.40 | 0.035 | 0.464 |
| Chemokine (C-C motif) ligand 8 | 4.03 | 0.005 | 0.412 | | Upk3b | 0.40 | 0.157 | 0.574 |
| Alox12 | 3.91 | 0.117 | 0.540 | | Wdr92 | 0.39 | 0.004 | 0.408 |
| Kcnk1 | 3.89 | 0.162 | 0.577 | | Lyz1 | 0.38 | 0.013 | 0.438 |
| D6Mit97 | 3.53 | 0.043 | 0.474 | | Msln | 0.36 | 0.079 | 0.508 |
| Igk-V5 | 3.52 | 0.032 | 0.463 | | Upk3b | 0.35 | 0.084 | 0.511 |
| Mia1 | 3.38 | 0.179 | 0.588 | | Muc16 | 0.34 | 0.078 | 0.507 |
| LOC384415 | 3.34 | 0.001 | 0.338 | | Chst4 | 0.32 | 0.034 | 0.464 |
| Igkv4-68 | 3.33 | 0.174 | 0.585 | | Bmp3 | 0.29 | 0.079 | 0.508 |
| Ighv1-62 | 3.31 | 0.020 | 0.446 | | Acta1 | 0.17 | 0.033 | 0.463 |
| Clic6 | 3.22 | 0.138 | 0.558 | |  |  |  |  |
| Cldn11 | 3.20 | 0.187 | 0.591 | |  |  |  |  |
| 1600029I14Rik | 3.04 | 0.173 | 0.584 | |  |  |  |  |
| A530020H22Rik | 2.96 | 0.109 | 0.534 | |  |  |  |  |
| C7 | 2.94 | 0.104 | 0.528 | |  |  |  |  |
| Igh-VJ558 | 2.94 | 0.072 | 0.500 | |  |  |  |  |
| Sox9 | 2.92 | 0.097 | 0.523 | |  |  |  |  |
| Igl-V1 | 2.92 | 0.169 | 0.582 | |  |  |  |  |
| Cyp2f2 | 2.88 | 0.292 | 0.663 | |  |  |  |  |
| Bbox1 | 2.80 | 0.178 | 0.587 | |  |  |  |  |
| Iyd | 2.79 | 0.256 | 0.638 | |  |  |  |  |
| LOC383196 | 2.74 | 0.110 | 0.535 | |  |  |  |  |
| LOC232065 | 2.74 | 0.014 | 0.438 | |  |  |  |  |
| Pcp4l1 | 2.74 | 0.238 | 0.625 | |  |  |  |  |
| Lrtm1 | 2.73 | 0.315 | 0.679 | |  |  |  |  |
| Cadherin-related family member 3 | 2.68 | 0.194 | 0.596 | |  |  |  |  |
| LOC637785 | 2.63 | 0.012 | 0.438 | |  |  |  |  |
| LOC383196 | 2.59 | 0.096 | 0.523 | |  |  |  |  |
| IGHV1S35_M12376_Ig_heavy_variable_1S35_13 | 2.52 | 0.047 | 0.476 | |  |  |  |  |
| Acsbg1 | 2.52 | 0.168 | 0.582 | |  |  |  |  |
| Slc40a1 | 2.49 | 0.207 | 0.604 | |  |  |  |  |
| Acpp | 2.45 | 0.143 | 0.562 | |  |  |  |  |
| Sectm1b | 2.44 | 0.194 | 0.596 | |  |  |  |  |
| Ubd | 2.43 | 0.019 | 0.445 | |  |  |  |  |
| Bex4 | 2.40 | 0.165 | 0.580 | |  |  |  |  |
| Fbp2 | 2.37 | 0.328 | 0.687 | |  |  |  |  |
| Ddit4l | 2.36 | 0.180 | 0.588 | |  |  |  |  |
| 2010001J22Rik | 2.36 | 0.201 | 0.600 | |  |  |  |  |
| Cxcl13 | 2.35 | 0.041 | 0.471 | |  |  |  |  |
| Gja1 | 2.33 | 0.143 | 0.561 | |  |  |  |  |
| Sult1c2 | 2.31 | 0.202 | 0.601 | |  |  |  |  |
| Lrp2 | 2.29 | 0.387 | 0.722 | |  |  |  |  |
| IGKV2-137_AJ231263_Ig_kappa_variable_2-137_15 | 2.27 | 0.083 | 0.511 | |  |  |  |  |
| LOC385291 | 2.26 | 0.218 | 0.611 | |  |  |  |  |
| Bex2 | 2.25 | 0.183 | 0.590 | |  |  |  |  |
| Slc27a2 | 2.23 | 0.226 | 0.615 | |  |  |  |  |
| BC038167 | 2.23 | 0.154 | 0.571 | |  |  |  |  |
| Nt5e | 2.22 | 0.192 | 0.594 | |  |  |  |  |
| LOC207685 | 2.20 | 0.100 | 0.525 | |  |  |  |  |
| LOC381284 | 2.17 | 0.124 | 0.544 | |  |  |  |  |
| Mmd2 | 2.17 | 0.140 | 0.559 | |  |  |  |  |
| Sh3gl2 | 2.16 | 0.148 | 0.566 | |  |  |  |  |
| IGHV1S120_AF025443_Ig_heavy_variable_1S120_8 | 2.16 | 0.042 | 0.474 | |  |  |  |  |
| Lrrc48 | 2.16 | 0.214 | 0.608 | |  |  |  |  |
| IGKV8-31_AJ235957_Ig_kappa_variable_8-31_3 | 2.13 | 0.017 | 0.445 | |  |  |  |  |
| IGKV12-98_AJ235949_Ig_kappa_variable_12-98_12 | 2.12 | 0.205 | 0.603 | |  |  |  |  |
| Sfrp1 | 2.11 | 0.085 | 0.511 | |  |  |  |  |
| IGLC2_J00595_Ig_lambda_constant_2_14 | 2.11 | 0.055 | 0.484 | |  |  |  |  |
| Spag6 | 2.09 | 0.314 | 0.678 | |  |  |  |  |
| Ppil6 | 2.08 | 0.310 | 0.674 | |  |  |  |  |
| LOC384419 | 2.07 | 0.058 | 0.485 | |  |  |  |  |
| Col6a5 | 2.06 | 0.344 | 0.697 | |  |  |  |  |
| Fcgr4 | 2.04 | 0.029 | 0.461 | |  |  |  |  |
| Nme7 | 2.04 | 0.434 | 0.751 | |  |  |  |  |
| BC021891 | 2.02 | 0.097 | 0.523 | |  |  |  |  |
| Dsg2 | 2.01 | 0.274 | 0.650 | |  |  |  |  |
| Hbb-b1 | 2.00 | 0.468 | 0.770 | |  |  |  |  |
| 1700024G13Rik | 2.00 | 0.190 | 0.593 | |  |  |  |  |

|  |
| --- |

^a^ Compared to the control group. Fold change limit 2.0.

^b^ Q-values are P-values corrected for multiple hypotheses using Benjamini-Hochberg false discovery rate.
